# Martini 3 as a Transferable Force Field for Lipopolysaccharide Parametrization

**DOI:** 10.1021/acs.jpcb.6c00050

**Published:** 2026-03-12

**Authors:** Gvantsa Gutishvili, Diane L. Lynch, James C. Gumbart

**Affiliations:** † School of Physics, 1372 Georgia Institute of Technology, Atlanta, Georgia 30332, United States; ‡ School of Chemistry & Biochemistry, Georgia Institute of Technology, Atlanta, Georgia 30332, United States

## Abstract

Lipopolysaccharides (LPS), as critical components of the outer membrane (OM) of Gram-negative bacteria, play essential roles in maintaining bacterial integrity and mediating environmental interactions. All-atom molecular dynamics (AA-MD) simulations provide detailed insights into LPS behavior at atomic resolution, but they remain computationally limited in accessing biologically relevant time scales. Coarse-grained (CG) models, such as Martini 3, offer a computationally efficient alternative while retaining sufficient accuracy for key biophysical properties. Although Martini 3 has been widely applied to proteins and phospholipids, only a few LPS models have been developed within this framework, limiting its utility for bacterial OM studies. To address this gap, we developed and validated CG parameters for LPS from multiple medically relevant pathogens, including *Escherichia coli* and *Salmonella enterica*, as well as two ESKAPE pathogens, *Klebsiella pneumoniae* and *Pseudomonas aeruginosa*. Our approach leverages the transferability of Martini parameters: we parametrized 57 unique disaccharide units using the Bartender tool, which automates CG-to-AA mapping and parametrization. These parameters were then combined and manually refined to accurately reproduce the complex dynamics of complete LPS molecules. We conducted extensive AA and CG simulations of asymmetric bilayers composed of phospholipids in the inner leaflet and LPS in the outer leaflet allowing detailed comparisons between the two for key structural and dynamic properties. The close agreement between the CG and AA simulations demonstrates the accuracy and robustness of our transferable parameter set, providing a valuable tool for simulating Gram-negative bacterial OMs at larger scales and longer time scales.

## Graphical abstract

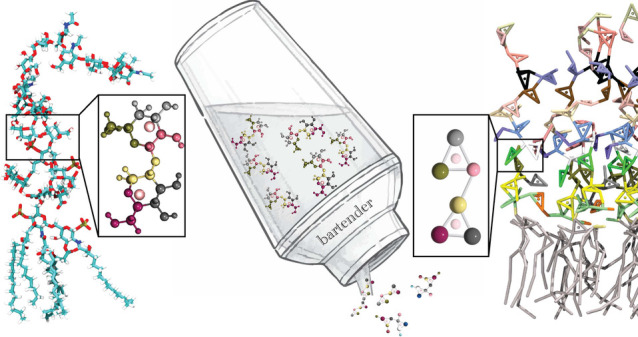

## Introduction

The OM of Gram-negative bacteria plays a crucial role in maintaining bacterial integrity, mediating interactions with the environment, and contributing to pathogenicity. A key component of the OM is LPS, a complex glycolipid that dominates the outer leaflet and provides a barrier against antibiotics, detergents, and host immune factors.^1,2^ LPS is composed of a trio of components: lipid A, a glycolipid whose acyl chains form the hydrophobic region of the outer leaflet of the OM, core oligosaccharide, which along with lipid A helps maintain the integrity of the OM, and the O-antigen, a repeating oligosaccharide of variable length. The latter is the hydrophilic outermost component, forms direct interactions with the bacterial environment, and is largely responsible for the antigenicity and antibody production in infected host cells.^3^ Significant variations in the composition of the O-antigen give rise to the large number of bacterial serotypes complicating therapeutic treatments. Due to their essential role in bacterial survival and virulence, understanding the structure and dynamics of LPS is critical for both basic research and the development of novel antimicrobial strategies.^4,5^

AA-MD simulations have provided valuable insights into bacterial membranes at atomic resolution.^6–11^ However, the large size and slow dynamics of LPS-rich membranes make it challenging to reach biologically relevant time scales using AA-MD alone.^12^ This is further complicated by the O-antigens, which are only rarely included in simulations. For example, Gao et al.^13^ performed all-atom simulations of LPS bilayers and further incorporated enterobacterial common antigen (ECA), finding it affects membrane packing, hydrophobic thickness, and area per lipid. To ameliorate this complication, a variety of coarse-graining approaches have been developed to meet the spatial and temporal challenges of AA systems.^14–17^ Of these the Martini force field has emerged as one of the most widely used.^15,18,19^

The Martini force field, initially focused on membrane simulations, was later extended to include membrane proteins, polysaccharides, glycolipids, as well as LPS.^15,20,21^ The use of Martini has proven to be very successful at achieving longer time-scales for larger system sizes than what is routinely possible with atomistic approaches.^15,19,22^ However, the widespread use of Martini has revealed significant force-field based limitations including the well-known overaggregation phenomena of both soluble and membrane proteins.^18,23,24^ Importantly, for LPS-based membrane systems the tendency to overestimate interactions between carbohydrates and glycolipids leads to artificial aggregation and poor transferability.^25,26^ These issues are especially problematic for LPS, whose extended oligosaccharide chains require accurate modeling of carbohydrate-carbohydrate and carbohydrate-lipid interactions. Moreover, limitations in lipid-based systems have also been reported, leading to a revision of lipid parameters.^27,28^

In order to address these issues, Martini 3^18,28^ has been developed, thereby improving the modeling of complex biomolecular systems and continues to be developed and validated for a variety of chemical and biological systems.^26,28–30^ Martini 3 addresses these shortcomings through a revised bead interaction matrix, canonical mapping scheme, and reparametrization.^18,26^ These advances make Martini 3 particularly well suited for simulating LPS-rich bacterial OMs where both phospholipids and complex glycans must be represented consistently. However, there are few existing CG models of LPS compatible with the new force field.^31,32^ In a recent study from Vaiwala and Ayappa,^31^ they constructed Martini 3 models for *E. coli* LPS outer membranes and validated bilayer structural properties. Additionally, Brandner et al.^32^ introduced Martini 3 and Martini 2 models for ReLPS, focusing on kinetic behavior and comparison with atomistic simulations. Despite these advances, existing CG LPS models remain tailored to specific chemotypes, limiting their applicability to the diverse LPS of other bacterial species.

To address this gap, we developed and validated new, transferable, Martini 3 bonded parameters for LPS from *E. coli* and *S. enterica*, as well as from selected ESKAPE pathogens including *K. pneumoniae* and *P. aeruginosa*. Our methodology is based on a building block approach, where we have parametrized a diverse set of disaccharides, combined them into the oligosaccharides/polysaccharides of the core/O-antigen components of LPS and finally attached these to lipid-A models using existing Martini 3 lipid open beta parameters, identical to a recently published parameter set,^28^ to generate multiple, chemically diverse bacterial strains. We parametrized 57 different disaccharides using a combination of automated matching to short semiempirical QM/MD simulations with the Bartender tool,^33^ followed by manual fine-tuning. Extensive CG simulations of asymmetric bilayers containing phospholipids in the inner leaflet and various LPS types in the outer leaflet were performed, generating more than 600 μs of total simulation time across 5 systems with two replicas each. Our simulations captured key membrane properties, including O-antigen spatial extension and tilt, membrane thickness, area per LPS, LPS diffusion constants, solvent-accessible surface area (SASA), compressibility and LPS entanglement coefficients demonstrating good agreement with extensive AA-MD results. Thus, we illustrate that our building-block approach is effective for modeling the diverse oligosaccharide components of LPS from Gram-negative bacteria.

## Methods

All CG simulations were performed using GROMACS 2023.1,^34^ employing the Martini 3.0.0.1 force field for lipids and solvents,^18,28^ along with custom polysaccharide parameters developed here and described more fully below. AA simulations were carried out using NAMD3 ^35^ using the CHARMM36 force field for LPS^36^ and lipids,^37^ along with the TIP3P water model.^38^

### System Preparation

To enable direct comparison between resolutions, we conducted extensive AA and CG simulations of asymmetric bilayers composed of phospholipids in the inner leaflet and LPS in the outer leaflet (see Figure 1). The bilayer composition and system size were matched closely between the AA and CG representations. AA systems were prepared using the CHARMM-GUI Bilayer Builder,^10,39^ while CG systems were constructed using the CHARMM-GUI Martini Maker^40^ for the lower leaflet and custom Python and Tcl scripts provided on Zenodo (DOI: 10.5281/zenodo.18009953) together with GROMACS tools.^41^ Specifically, gmx trajconv and gmx editconf were employed for the upper leaflet, and gmx solvate and gmx genion were used to solvate and ionize the resulting bilayers, yielding a composition closely matching the AA models. Each system contained 34 LPS molecules in the upper leaflet, with the lower leaflet composed of the phospholipids POPE (75%), POPG (25%), in line with previous studies.^6^ All systems were solvated in a 0.15 M monovalent salt solution (KCl), and the net negative charge of LPS molecules was neutralized by adding divalent Mg^2+^ and Ca^2+^ cations.

**Figure 1:**
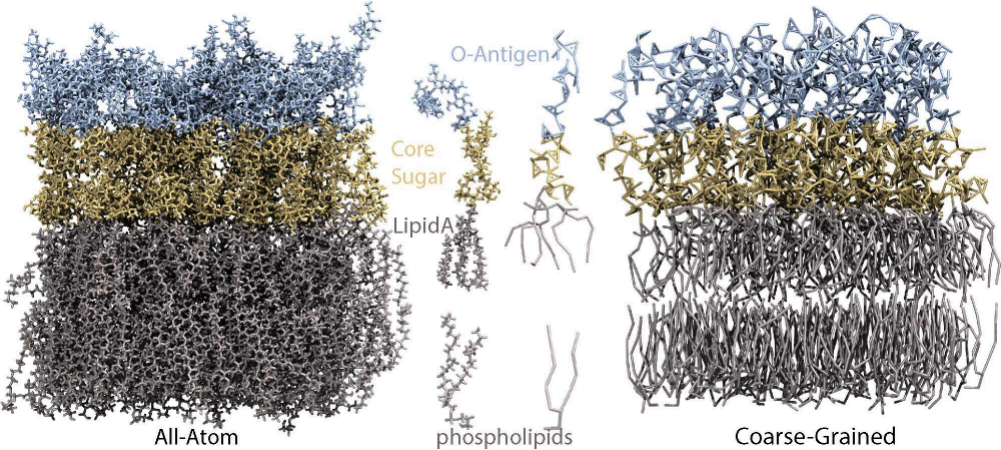
AA and CG representations of the O157 LPS membrane. Shown are snapshots of the bilayer in AA (left) and CG (right) simulations, with example LPS and phospholipid molecules highlighted in both representations. Phospholipids and Lipid A are in gray, core sugars in yellow, and O-antigens in cyan.

### CG Parameters

CG models of bacterial species specific LPS molecules were built by developing Martini 3-compatible parameters for disaccharides and combining these into appropriate core oligosaccharide O-antigen units. Parameters for the lipid A component were taken from Vaiwala and Ayappa;^31^ for the *S. enterica* serovar used here, these parameters were modified to allow for LPS with seven (instead of the more common six) aliphatic tails. An example of the AA-to-CG mapping used is illustrated in Figure 2 for the *E. coli* O157 LPS with the composition of the bacterial strains is listed in Table S1. The diversity of disaccharides required for the bacterial systems considered here is illustrated in Figure S1, where each LPS is rendered using symbol nomenclature for glycans (SNFG) format^42,43^ and clearly shows the number of sugar permutations required to build a complete LPS; here this amounted to 57 distinct disaccharides which are listed in Table S2.

**Figure 2:**
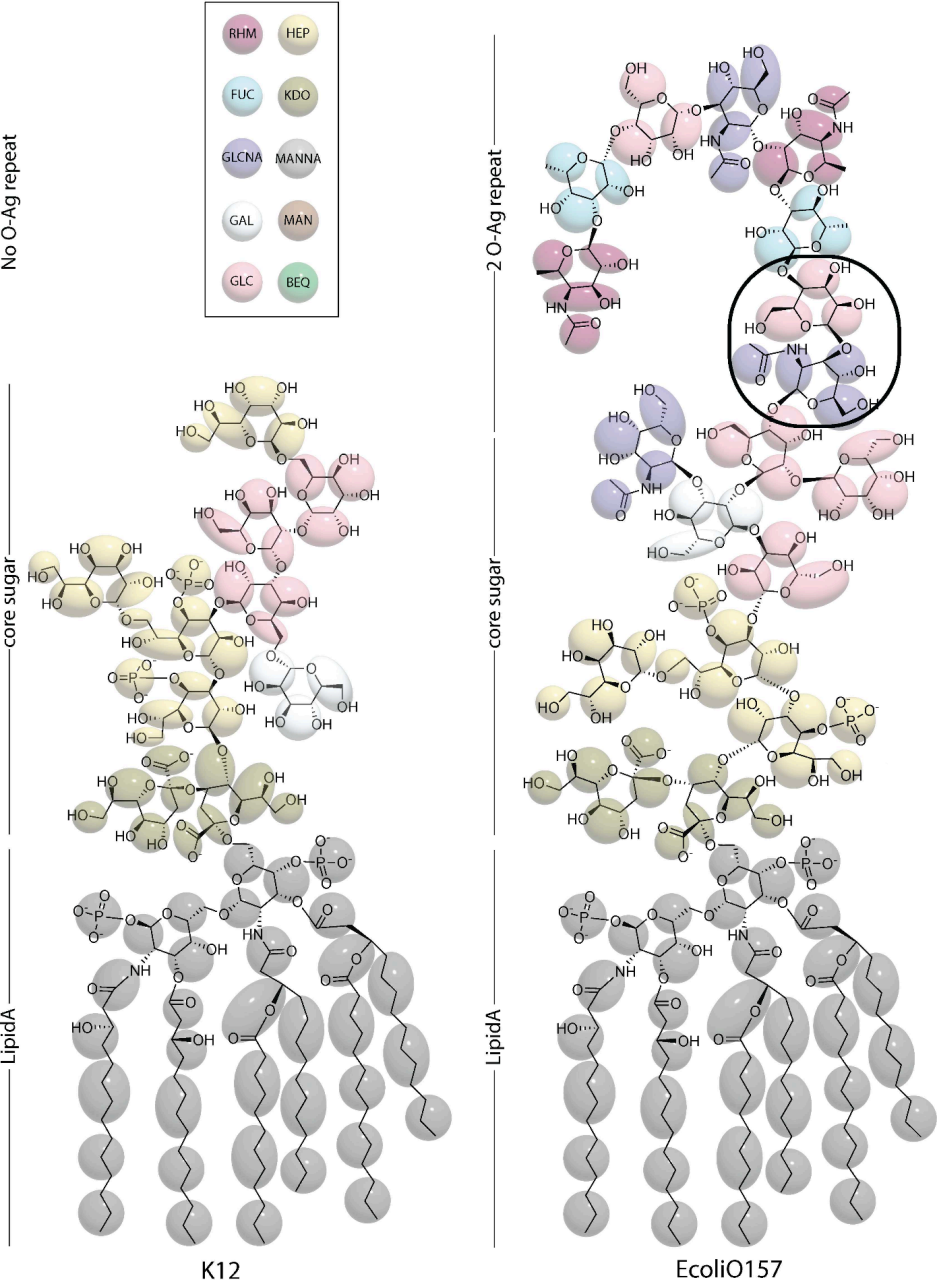
Structures of *E. coli* K12 and O157 LPS. The schematic highlights the details in core oligosaccharide and O-antigen composition in these two serotypes.^44,45^ Shaded areas depict Martini 3 beads with the gray beads representing the lipid A (ECLIPA), and other colors: red, yellow, cyan, olive, purple, light gray, white, brown, pink, green are Rhamnose (RHM), Heptose (HEP), Fucose (FUC), 3-deoxy-D-manno-oct-2-ulosonic acid (KDO), N-acetyl-d-glucosamine (GLCNA), N-acetyl-D-mannosamine (MANNA), Galactose (GAL), Mannose (MAN), Glucose (GLC), and Abequose (BEQ), respectively. The β-glucose (1 → 3) β-N-acetyl galactosamine pair is outlined using a black circle.

Specifically, each disaccharide was first mapped from their atomistic counterpart to CG beads, i.e., 2–4 heavy atoms, including covalently attached hydrogen atoms, are replaced with a single Martini 3 bead. Bead types and atom groupings were assigned according to the Martini 3 parametrization guidelines.^46^ An example disaccharide, the β-glucose (1 → 3) β-N-acetyl galactosamine present in the O-antigen of *E. coli* O157 LPS, is indicated with a circle in Figure 2, with explicit assignment of Martini 3 beads illustrated for both *E. coli* serotypes in Figure S2. Similar mappings for the other bacterial strains are indicated in Figure S3. Bonded parameters for each disaccharide were derived using Bartender,^33^ an open-source command-line tool written in Go that automates CG-parameter optimization. Bartender takes an atomistic structure and a mapping file defining Martini beads and bonded interactions, then performs semiempirical MD simulation, via the extended tight binding (xtb) program,^47^ or uses pregenerated trajectories to sample bond, angle, and dihedral distributions. These distributions are then fit to generate Martini 3-compatible bonded potentials, ensuring that the CG model accurately reproduces the conformational behavior observed in atomistic simulations. An example of the AA-to-CG mapping is provided in Figure S4 for the disaccharide β-glucose (1 → 3) N-acetyl galactosamine, with a comparison of the fit to QM results for the corresponding bonds and angles given in Figures S5 and S6. In the present parametrization, bonded interactions between disaccharide units were described using bond and angle potentials only. No explicit torsion (dihedral) potentials were included, as the conformational behavior observed in the atomistic reference simulations was adequately reproduced without the need for additional terms.

Here we have employed the GFN2-xTB functional^48^ for 2–10 ns of QM/MD. With the necessary disaccharides parametrized, these were joined to produce the complete core oligosaccharide and O-antigen LPS structures corresponding to the *E. coli* O157 (EcoliO157), *S. enterica* O4 (SEnO4), *K. pneumoniae* O1-2 (KPO1-2), and *P. aeruginosa* O5a (PAO5a) serotypes with two O-antigen repeats each. During assembly of the full LPS molecules, all neighboring disaccharide pairs present in the target sequences were explicitly parametrized at the building-block level. For example, for a sequence sugar_1_-sugar_2_-sugar_3_-sugar_4_, bonded parameters were derived independently for sugar_1_-sugar_2_, sugar_2_-sugar_3_, and sugar_3_-sugar_4_. This ensured that each local glycosidic environment appearing in the final LPS was directly represented by a corresponding disaccharide parameter set. For each glycosidic linkage, one bond potential and four associated angle potentials (two defined on each side of the bond) were included. No explicit dihedral potentials were introduced. For each disaccharide, a CG mapping was defined by grouping atoms into Martini beads similar to LPS mapping. Bartender was then used to optimize the bonded parameters (bond and angle terms) for the mapped topology. Bead types for the final LPS models were assigned according to standard Martini 3 rules. Assembly of full-length LPS molecules was performed using custom Python scripts, which preserved bead identities and bonded connectivity with minimal additional manual modification at the polysaccharide level. To illustrate this workflow, we provide example scripts on Zenodo (DOI: 10.5281/zenodo.18009953) that show how individual disaccharide parameters can be combined into larger oligosaccharides and subsequently incorporated into complete CG membranes. These examples are intended to serve as a practical guide for reproducing the parametrization strategy. Parameter validation was performed by comparing structural features, such as O-antigen tilt, end-to-end distance, membrane thickness, area per LPS, SASA, LPS diffusion constants, compressibility modulus and LPS entanglement coefficients with benchmarks from AA simulations.

### Simulation Protocol

Two replicas were simulated for both the AA and CG systems, summarized in Table 1. The CG simulation protocol began with 200 steps of soft-core minimization, required for single-precision GROMACS, followed by a staged equilibration: 4 ps of MD with a 1 fs time step, gradually increased to 5 fs for 10 ps and then to 10 fs for 60 ps. Production simulations were carried out in the NPT ensemble at 310 K and 1 bar using a velocity-rescale thermostat and a C-rescale barostat with pressure and temperature coupling constants of 4 and 1 ps, respectively. Nonbonded interactions were computed using the reaction-field method for electrostatics with a relative dielectric constant of 15 and a cutoff of 1.1 nm for both Coulomb and van der Waals interactions. van der Waals interactions employed a potential-shift Verlet scheme, and the Verlet cutoff scheme with a buffer tolerance of 0.005 kJ mol^–1^ ps^–1^ was used. Semi-isotropic pressure coupling was applied with a compressibility of 3 × 10^–4^ bar^–1^, and bond constraints were enforced using the LINCS algorithm with an eighth-order expansion and a 90° warning angle. The final production simulations used a 10 fs time step. Tests using a 20 fs time step led to numerical instability for these LPS systems, primarily due to the presence of “tiny” beads in the carbohydrate representation. AA protocol began with 2000 steps of energy minimization, followed by 40 ns of equilibration with a 2 fs time step while gradually decreasing spatial restraints. Production simulations were performed in the NPT ensemble at 310 K and 1 bar using Langevin dynamics for temperature control with a damping coefficient of 1 ps^–1^. Pressure was maintained using a Langevin piston barostat with semi-isotropic coupling, an oscillation period of 0.3 ps, and a damping time of 0.15 ps. Long-range electrostatics were treated with the particle-mesh Ewald (PME) method using a real-space cutoff of 12 Å and a particle-mesh Ewald (PME) grid spacing of 1.0 Å, while van der Waals interactions were smoothly switched off between 10 and 12 Å using a force-switching function. Hydrogen mass repartitioning enabled the use of a 4 fs time step during production runs.^49^

**Table 1:** Summary of Simulated Systems^a^

System	LPS Type	Force Field	Simulation Length
I	KPO1-2	CHARMM36	10 μs
II		MARTINI3	50 μs
III	PAO5a	CHARMM36	10 μs
IV		MARTINI3	50 μs
V	EcoliO157	CHARMM36	10 μs
VI		MARTINI3	50 μs
VII	SEnO4	CHARMM36	10 μs
VIII		MARTINI3	50 μs
IX	EcoliK12	CHARMM36	10 μs
X		MARTINI3	50 μs

a Each system was simulated in two replicas. In all systems, the inner leaflet consists of 75% POPE and 25% POPG, while the outer leaflet is composed entirely (100%) of LPS molecules.

### Analysis

For each system, the two independent replicas were analyzed, and averages are reported together with standard deviations to indicate variability. Visualization and figure rendering were carried out in VMD.^50^ The first 10% of each production trajectory was omitted to remove initial equilibration artifacts.

Structural properties of the O-antigen were assessed by measuring glycan tilt relative to the membrane normal and by calculating end-to-end distances of the polysaccharide chains. Bilayer thickness was computed from the average phosphate-phosphate distance across the two leaflets using gmx density. Area per LPS was obtained from the projected bilayer area normalized by the number of lipids in the outer leaflet. Each membrane was centered on its geometric center prior to number density calculations.

SASA was calculated for each LPS for each frame of the trajectory using VMD’s measure sasa and gromacs sasa command with a probe radius of 1.4 Å, corresponding to a water molecule. The area compressibility was computed from the fluctuations of the membrane area during the production trajectories. For every frame, the instantaneous membrane area was obtained from the simulation box dimensions in the membrane plane as *A*_*i*_ = *L*_*x*_ *L*_*y*_. The resulting time series {*A*_*i*_} was used to compute the mean membrane area A̅ and the variance of the fluctuations ${\sigma_{A}}^{2}$ . Following the standard fluctuation–dissipation relation, the area compressibility parameter was calculated as $\kappa =k_{B}T\mathrm{A̅}/{\sigma_{A}}^{2}$ . This definition quantifies the relative magnitude of membrane area fluctuations providing a measure of the membrane’s sensitivity to in-plane deformation. Diffusion coefficients were calculated from the mean-squared displacement (MSD) of LPS molecules, using linear fits to the MSD curve, following the procedure of Balusek et al.^49^

AA and CG systems were constructed using different CHARMM-GUI modules appropriate for each resolution. Although membrane composition, leaflet asymmetry, box dimensions, lipid counts, and ionic conditions were kept identical across resolutions, given the slow relaxation of LPS-rich membranes, there is likely some remaining bias due to the initial system set up. In all systems, area per lipid, membrane thickness, and O-antigen extension relax away from their initial values and reach stable plateaus prior to the analysis window. All reported averages were therefore computed from equilibrated trajectory segments.

### Quantification of LPS-LPS Entanglement

To quantify the degree of topological interweaving between O-antigen chains, we computed the Gaussian Linking Number (GLN) for every pair of LPS molecules in the CG and AA membrane simulations. The GLN is a classical topological invariant that measures the average signed winding of two curves around one another.^51,52^ Low GLN values indicate chain pairs that are either noncontacting or do not wrap around each other, whereas larger GLN values reflect an increased degree of mutual wrapping, corresponding to a greater number of interchain loops. For each trajectory, we assigned a fixed chain identifier to every LPS molecule and extracted the spatial coordinates of its LPS backbone beads (excluding the lipid A core). Each LPS chain in a given frame was represented as a piecewise-linear curve defined by its backbone sugar center-of-geometry coordinates.

For a given pair of chains, the GLN was computed numerically using a discretized double line-integral approximation. Each chain was decomposed into sequential line segments, defined by the vector between the center of geometry of adjacent sugar moieties, and we evaluated the pairwise contribution

1 $$GLN\left(C_{1},C_{2}\right)=\frac{1}{4\pi }\underset{i,j}{\sum }\frac{R_{ij}\cdot \left(d_{i}\times d_{j}\right)}{{∥R_{ij}∥}^{3}}$$

where **d**_*i*_ and **d**_*j*_ are the direction vectors of segments on chains *C*_1_ and *C*_2_ and **R**_*ij*_ is the vector connecting the midpoints of the two segments.^53^ A short-range regularization parameter (ε = 0.03 nm) was used to avoid numerical divergence when segment midpoints are extremely close. GLN values were computed for every pair of LPS chains for trajectory frames sampled every 100 steps, accumulated over the full production trajectory, and averaged to obtain a time-averaged GLN matrix for each system. Each element of the GLN matrix represents the time-averaged GLN between a pair of LPS chains, quantifying how strongly the two chains wrap around or thread through one another over the trajectory. Values close to zero indicate little or no topological coupling, whereas larger absolute values correspond to stronger mutual entanglement involving multiple wraps or loops. To characterize the distribution of entanglement strengths, we analyzed the absolute values of the off-diagonal GLN matrix elements. Probability density functions (PDFs) report the relative frequency of pairwise entanglement magnitudes, highlighting the most probable |GLN| values sampled in the simulations. In addition, PDFs pooled across all strains provide a global view of the entanglement landscape, revealing systematic differences in the typical strength and spread of LPS-LPS entanglement between atomistic and CG representations independent of strain-specific features. Complementary cumulative distribution functions (CDFs) show the fraction of chain pairs with entanglement strengths below a given |GLN| threshold, enabling direct comparison of overall entanglement across systems and resolutions. All analyses were performed using Python, MDAnalysis,^54^ NumPy,^55^ and custom routines implementing the discretized Gauss linking integral.

## Results

### CG Simulations Accurately Represent Key Biologically Relevant Features

#### The LPS Tilt Angles and Extension of LPS Are in Good Agreement between Simulations at Different Resolutions

The O-antigen component of LPS plays a distinct role in the surface presentation of bacteria and is central in the immunological response upon infection. Aside from its innate antigenic activity, Domínguez-Medina et al.^9^ have shown that LPS of sufficient length can occlude surface proteinaceous antigenic OM components, and it is the balance of their flexibility and extent that governs the transient opening of a path for antibodies to successfully recognize surface antigens. Therefore, extent and flexibility are critical properties of any LPS model and metrics that encode these features include the O-antigen tilt angles as well as the end-to-end distance of the oligosaccharide units.

To assess how well the CG parameters reproduce these key physical behaviors of LPS, we compared important structural characteristics between AA and CG simulations. The flexibility of LPS can be influenced by its tilt relative to the membrane normal (*z*-axis), as shown in Figure 3 for both the orientation of the O-antigens as well as the acyl chains of lipid A. O-antigen tilt angles (α) are measured relative to the membrane normal using the vector formed from the outer core sugar residues to the terminal O-antigen residues. The specific beads/residues for the CG/AA simulations are system specific and are given as follows. For the EcoliO157 system, the terminal core sugars were defined as residues 4 and 5, and the end of the O-antigen as residues 18 and 19. In the SEnO4 system, the reference point was the center of geometry (COG) of residues 4 and 5, and the terminal residues were 20 and 21. For the KPO1-2 system, the COG reference was based on residues 4 and 7, with the O-antigen end point defined as residues 16 and 17. In the PAO5a system, the terminal core sugar reference was the COG of residues 4 and 5, while residues 15 and 16 marked the O-antigen end point. Small oscillations are observed in the O-antigen tilt distributions (Figure 3b, top panel), similar to that reported by Vaiwala and Ayappa.^31^ Overstructuring of CG results relative to their AA counterparts was also observed by Loose et al.^56^

**Figure 3:**
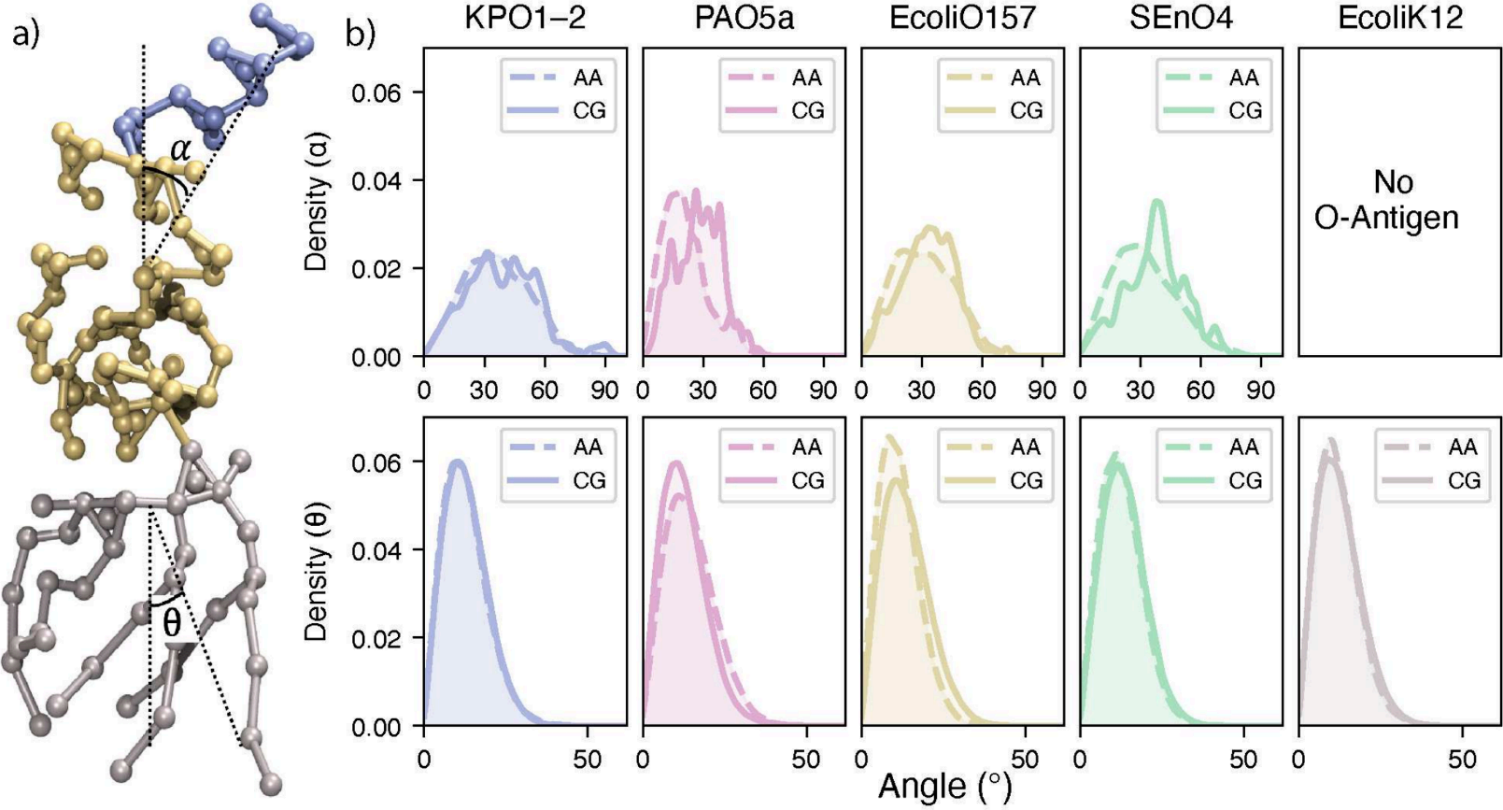
LPS tilt angles. (a) Graphical representation of LPS illustrating the tilt angles. The blue, gold, and gray beads are the O-antigen, core sugar, and lipid A species, respectively. (b) Comparison of AA and CG tilt angle distributions for the O-antigen tilt angle (α) and the lipid tilt angle (θ). Corresponding per-replica angle distributions for all serotypes are provided in the Supporting Information (Figure S7).

The orientation of the lipid acyl tails in each bacterial system was monitored using the tilt angle θ. As seen in Figure 3b, the orientation of the O-antigens (upper panel, α) is quite broad compared to the tilt of the lipid A moiety (lower panel, θ), reflecting the increased flexibility of the O-antigen regions of LPS relative to the lipid A component. As expected, since the lipid A component is the same in all the presently simulated systems and is taken from Vaiwala,^31^ the distributions are quite similar and centered about 10^*o*^ as reported earlier.^31^ Overall the orientations of the LPS in the present CG models is in good agreement with the AA results.

Extension was measured as the end-to-end distance between the last sugar of the O-antigen and the reference sugars from the core sequence, with the specific residues defined above (Figure 4). The Martini CG results display a wide distribution of distances, which is shifted by ∼5–10 Å to smaller values than their AA counterparts.

**Figure 4:**
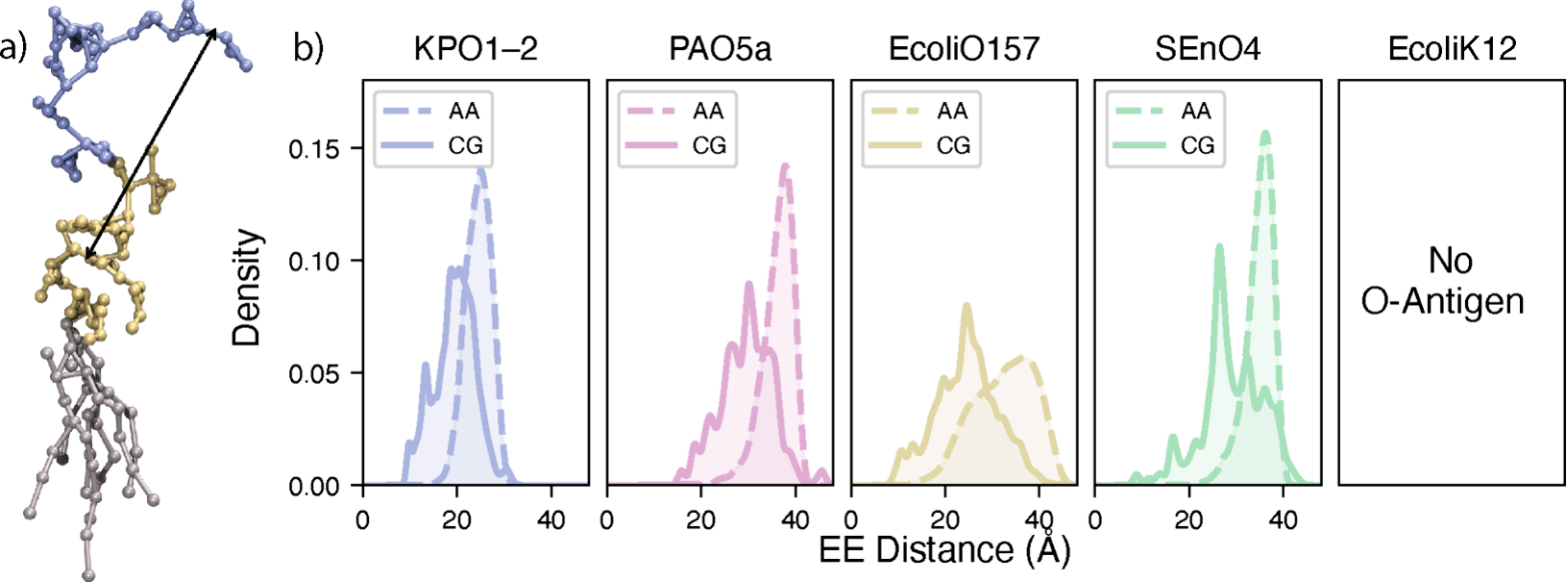
End-to-end LPS distances. (a) Graphical representation of KPO1-2 LPS, where the blue, gold, and gray beads are the O-antigen, core sugar, and lipid A species, respectively. The end-to-end distance is rendered with a black line. (b) Comparison of the end-to-end distance distributions for LPS serotypes in the CG and AA simulations. Corresponding per-replica end-to-end distance distributions for all serotypes are provided in the Supporting Information (Figure S8).

#### Membrane Thickness Is Comparable between the AA and CG Representations

To evaluate how well the CG model captures membrane structural features, we compared the bilayer thickness between CG and AA simulations in Figure 5. Thickness was measured as the average distance between the phosphorus atoms (or D-beads in CG) in the upper (lipid A) and lower (phospholipid) leaflets. For each system, the membrane thickness probability density functions are illustrated, enabling direct comparison of leaflet spacing and overall bilayer dimensions across resolutions and providing a direct way to assess how closely the CG model reproduces the physical membrane structure observed in higher-resolution AA simulations. Across all serotypes, the measured thickness values show approximate agreement between the AA and CG results, with significant overlap of the AA and CG distributions. CG membranes reproduce the relative ordering of membrane thickness among serotypes while systematically yielding slightly larger values than AA; however, given the broad distributions, these results indicate that the CG parametrization accurately reflects bilayer packing and leaflet spacing.

**Figure 5:**
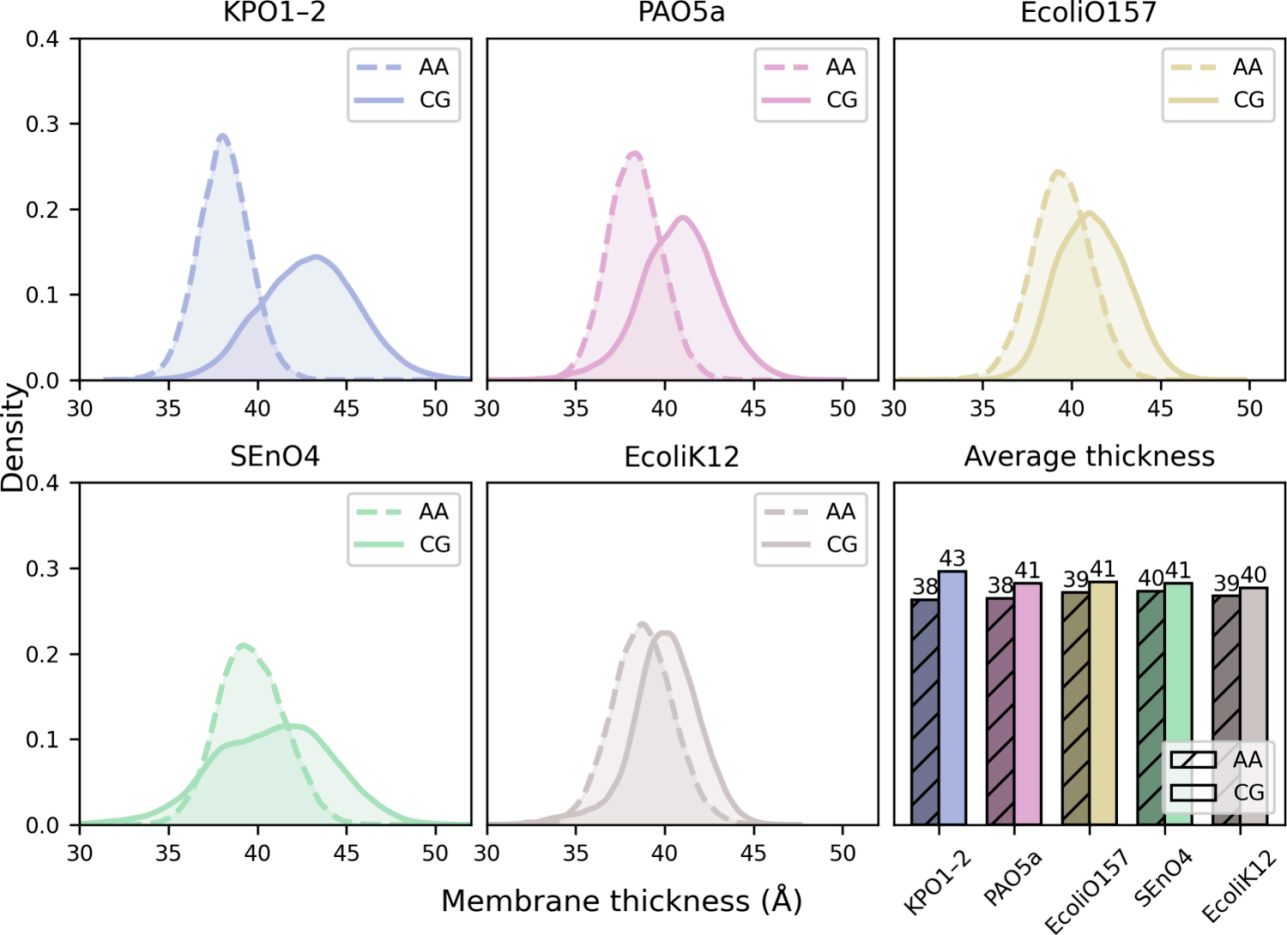
Membrane thickness of LPS-containing bilayers in AA and CG simulations. The bottom-right panel summarizes the mean membrane thickness for each serotype, with averages reported on the top of the bar. Corresponding per-replica membrane thickness probability distributions for all serotypes are provided in the Supporting Information (Figure S9).

Moreover, we have computed the area per lipid (APL), given in Figure 6, with the time-series provided in Figure S11. The present CG models generally produce lower APL values than the AA models, with several CG serotypes producing values ∼10% smaller (e.g., SEnO4 and KPO1-2) while the EcoliO157 and PAO5a distributions overlap. This general trend is in agreement with earlier study reporting tighter packing in CG membranes, relative to the corresponding AA membranes, with O-antigen units.^31^ Notably, Brandner et al.^32^ report a deviation from the AA-MD area per lipid for Martini 3.0 that is within ∼9% for ReLPS, a deep rough LPS model.

**Figure 6:**
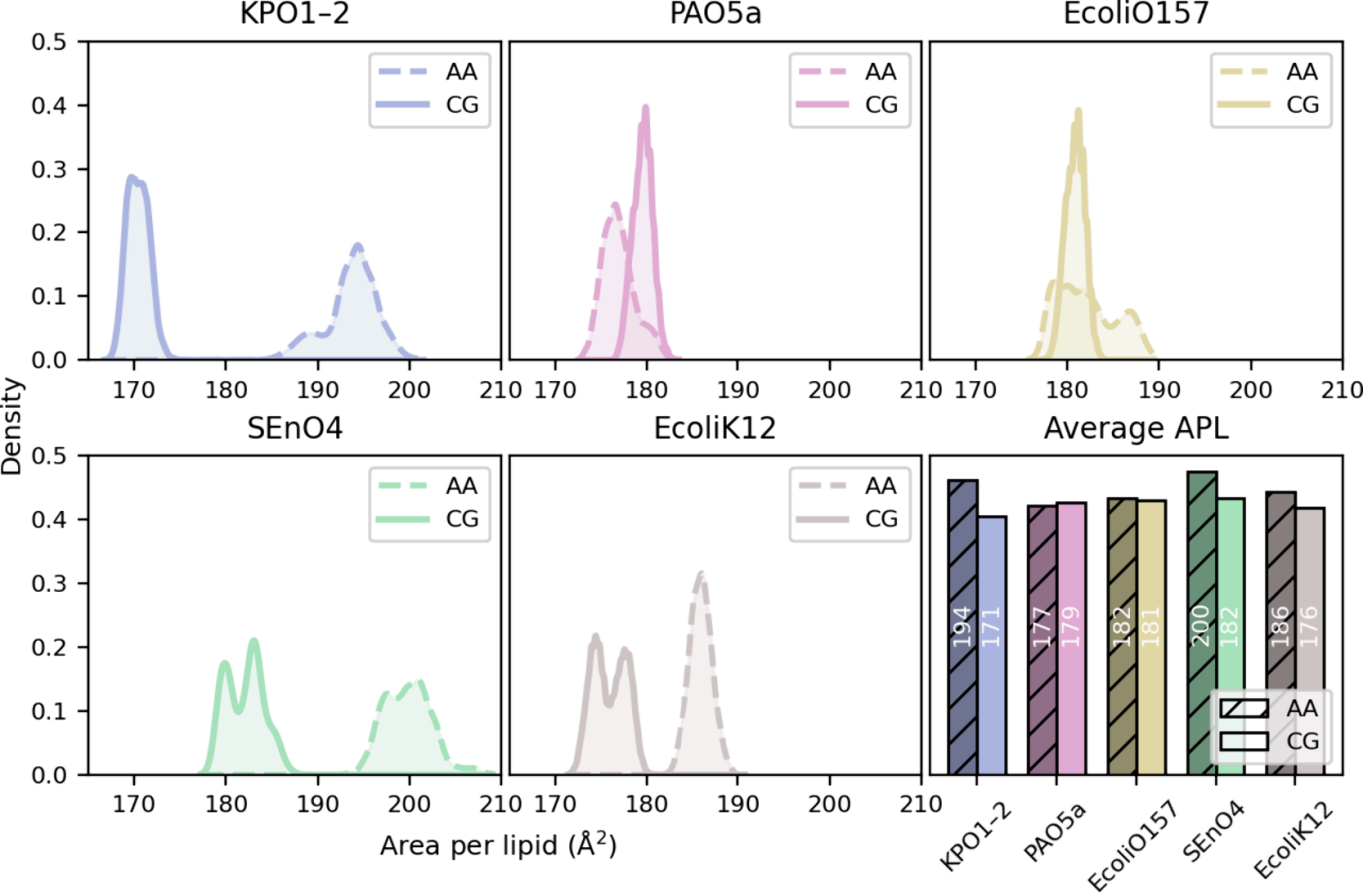
Area per LPS (APL) across all serotypes from CG and AA simulations. For each system, APL density distributions averaged over two independent replicas are shown, illustrating variability within and between resolutions. The bar plot in the lower right summarizes the mean APL values, averaged over both time and replicas, highlighting systematic differences between AA and CG membranes. Corresponding per-replica APL distributions for all serotypes are provided in the Supporting Information (Figure S10).

APL and bilayer thickness are geometrically coupled observables, and an inverse relationship between these quantities is generally expected: tighter lateral packing (smaller APL) leads to increased extension along the bilayer normal. Across most systems studied here, we observe this qualitative inverse relationship when comparing AA and CG models. In particular, systems that display reduced APL in the CG representation relative to AA typically exhibit correspondingly increased membrane thickness. PAO5 represents a mild exception, where the CG APL is larger than in AA while the thickness is also slightly increased; however, this difference in APL is small.

#### Membrane Area Compressibility Is Consistent between AA and CG Representations

In addition to local structural observables, we quantified the in-plane mechanical response of the LPS membranes through an area compressibility parameter κ, obtained from the fluctuations of the bilayer area (see Methods). For each serotype and resolution, κ was computed from the time series of instantaneous membrane areas, with the mean and standard deviations reported in Figure S16. Overall, the CG and AA simulations yield area compressibility values of the same order of magnitude, with CG membranes generally exhibiting larger κ values, indicative of reduced area fluctuations than their atomistic counterparts (Figure S16) for all systems except SEnO4, where CG κ values are slightly lower. This deviation is most likely influenced by differences in the number and organization of Lipid A tails in the *Salmonella* membrane. Taken together, these results indicate that the Martini 3 models not only reproduce the average bilayer structure but also capture at a semiquantitative level the overall in-plane mechanical stiffness of LPS-rich OMs. It is noteworthy that area compressibility has been reported for several atomistic force fields, as well as various CG models,^57^ highlighting the large variability in this parameter depending on the force field.

#### Membrane Density Distribution Is Comparable between These Two Representations

To further validate the accuracy of the CG model, we compared membrane density distribution profiles obtained from CG and AA simulations in Figure 7. Density profiles were calculated along the membrane normal (*z*-axis) by averaging atomic or bead number over the course of the simulation, reported in units of Å^–3^. These profiles capture the spatial organization of key membrane components, including lipid A, core sugar, and O-antigen chains. The resulting distributions revealed similar peak positions and overall shapes between the two models, indicating that the CG representation preserves essential structural features of membrane organization, such as bilayer asymmetry and leaflet separation.

**Figure 7:**
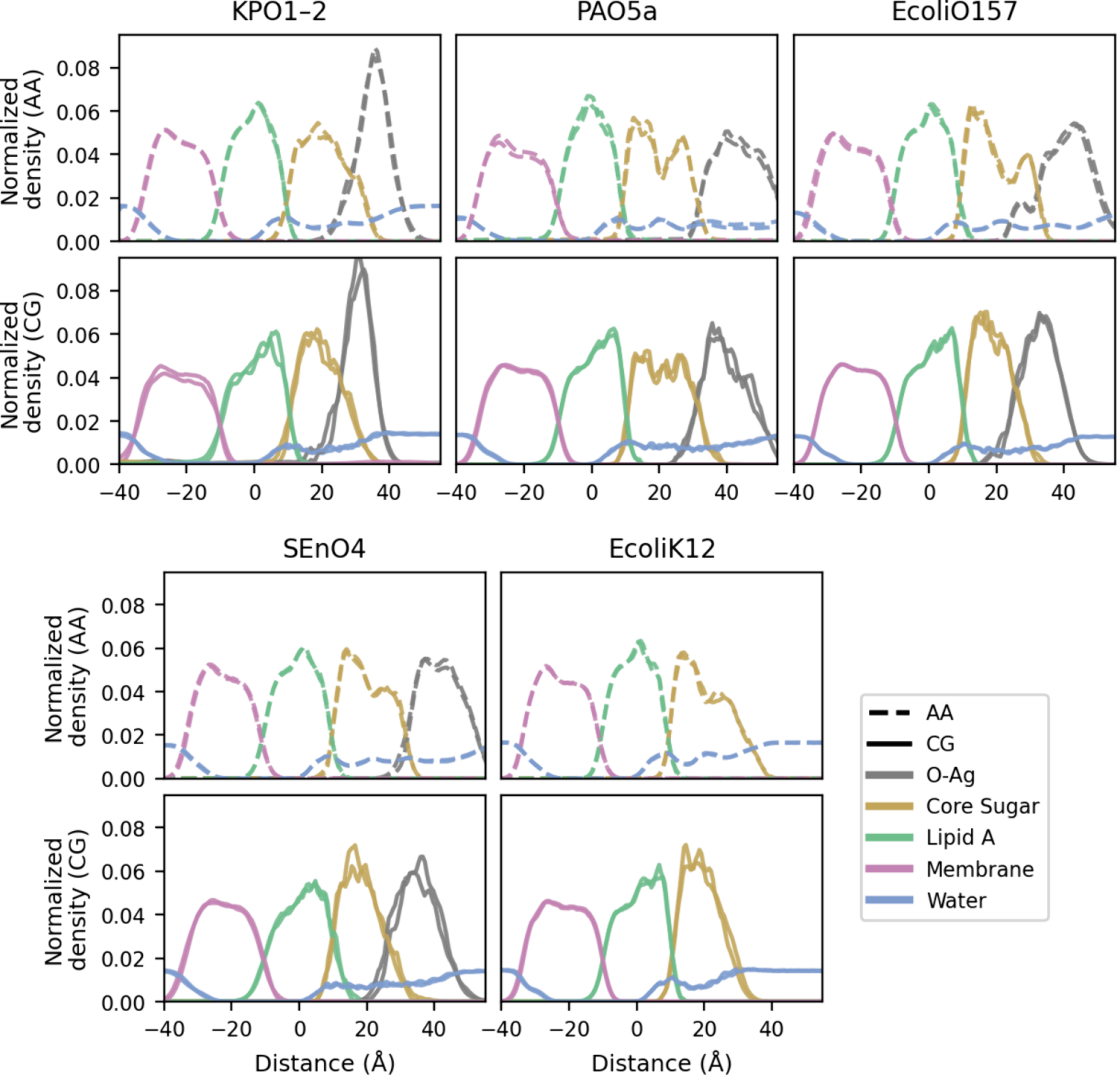
Normalized number density distributions for AA and CG systems. For each bacterial system the upper/lower panels report AA/CG results, respectively. Densities are provided for the aqueous phase (water/blue), the lipid A (green), the core oligosaccharides (yellow), the O-antigens (brown), and the phospholipids of the inner leaflet (pink) for each replica. The membrane is oriented with the outer leaflet to the right and inner leaflet to the left.

#### Divalent Cation Distributions Are in Good Agreement between the AA and CG Representations

Divalent cations play a prominent role in the structure and dynamics of Gram-negative OMs.^58–60^ The presence of multiple negatively charged phosphate groups in the lipid A core region, as well as phosphate- and carboxylate-substituted sugars, are neutralized by the presence of divalent cations. In addition, these ions act as a bridge between LPS molecules^11,60^ helping to maintain the structural integrity of the OM.

Number density distributions were calculated along the membrane normal for the phosphate and carboxylate groups, as well as the divalent cations (Ca^2+^ and Mg^2+^). These distributions (Figure S14) provide insight into the spatial organization of electrostatic interactions within the LPS-rich OM. In the AA simulations the divalent cations are localized near the negatively charged groups as expected. It is noteworthy that in the Martini force field, divalent cations are represented with a single bead representing both the ion as well as the first hydration shell; therefore specific, charge reinforced hydrogen bonding interactions will be lacking and a less localized behavior can be expected. In the present case we observe distributions that appear broader and less well-defined. The general agreement between CG and AA representations highlights the ability of the Martini 3 parameters to capture these essential electrostatic features.

#### LPS-LPS Entanglement from Gaussian Linking Analysis

The Gaussian Linking Number (GLN) analysis revealed consistent and reproducible patterns of O-antigen entanglement across all five LPS-containing systems. For each strain, we computed the distribution of pairwise |GLN| values over the production trajectories for each replica and combined the data. The full probability density functions (PDFs) and cumulative distribution functions (CDFs) obtained from the AA and CG simulations are compared in Figure 8.

**Figure 8:**
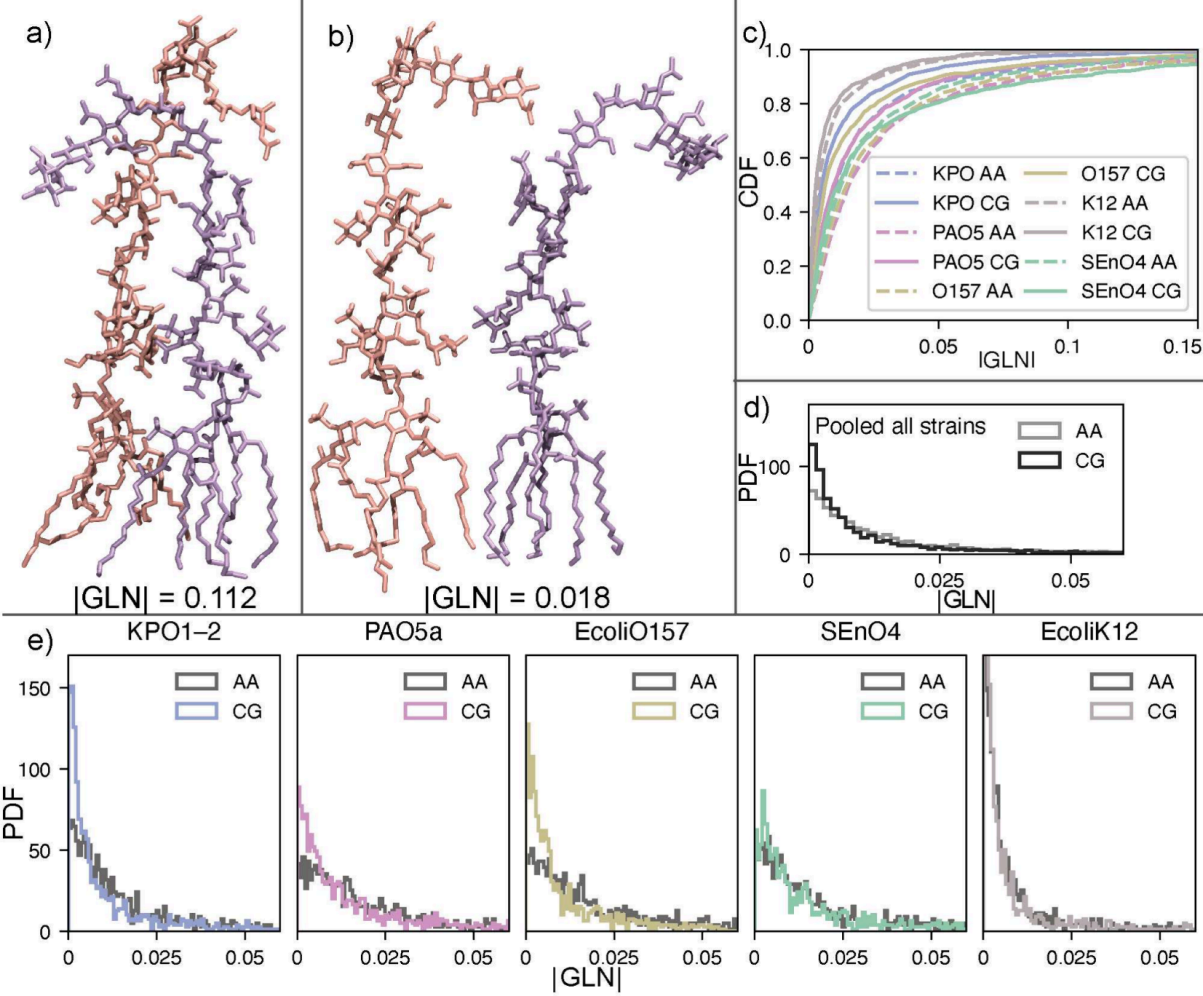
Topological coupling of LPS chains quantified by the Gaussian Linking Number (GLN). (a,b) Visual illustration of low and high GLN values, reflecting weak versus strong mutual wrapping between polysaccharide chains. (c) Cumulative distribution functions (CDFs) comparing the prevalence of weak and strong entanglement across systems and between AA and CG resolutions. (d) Probability density functions (PDFs) pooled across all strains, providing an overall comparison of typical entanglement magnitudes. (e) Per-system GLN distributions, reporting the time-averaged strength and variability of pairwise entanglement in AA and CG simulations.

Overall, the AA and CG GLN distributions are in excellent agreement, with the exception of an increased probability at low GLN values observed in the CG models of KPO1-2, PAO5a, and EcoliO157. For each serovar, the density profiles (Figure 8e) exhibited highly similar shapes at both resolutions, indicating that the CG model faithfully reproduces the degree of LPS-LPS topological interweaving observed in the atomistic simulations. As expected for flexible O-antigen chains in a fluid membrane, the GLN values remained small, reflecting that the chains may approach and pass near one another but do not form loops or wrap around each other. Although we have restricted our simulations to systems with only two O-antigen repeat units, depending on serotype and strain the LPS molecules may contain tens or more repeat units, and in such cases entangling may be much larger.

To complement the per-strain analysis, we generated a combined GLN distribution plot that includes all LPS systems (Figure 8d). The density curves highlight clear biological differences between strains: systems with longer and more flexible O-antigens (KPO1-2, EcoliO157, SEnO4, and PAO5a) exhibit broader distributions indicating an increased propensity for chain–chain winding. In contrast, the system with shorter saccharide chains (EcoliK12) display narrower distributions. Importantly, these strain-dependent trends were reproduced at both resolutions. Finally, the cross-system CDF comparison (Figure 8c) further underscores the quantitative agreement between AA and CG. In each strain, the AA and CG CDF curves overlap across nearly the entire |GLN| range. This indicates that not only the typical linking behavior but also rare high-linking events are consistently captured by the CG model.

Taken together, these results show that the present building-block approach to designing Martini 3 CG representations of the O-antigen repeat units preserves both the distribution and the relative ordering of LPS topological entanglement across strains, supporting its suitability for mesoscale analysis of O-antigen organization and chain–chain interactions.

#### SASA Is Shifted Relative to AA-MD

In addition to membrane specific structural parameters, we have also evaluated the LPS SASA using the present Martini 3 model and compared these results to the AA-MD simulations in Figure 9 (see Figure S13 for the time series). Aside from KPO1-2, the present results display a systematic shift to larger area relative to AA values. For example EcoliK12 is shifted by ∼17% and SEnO4 by ∼11%, EcoliO157 by ∼14% and PAO5a by ∼16%. Consistent with previous work,^31,32^ we observe higher SASA in CG simulations compared to AA. However, the magnitude of the CG-AA difference is notably larger in most of our systems. This difference may be partly explained by the greater overall length of our modeled LPS molecules, particularly in the O-antigen region, where accumulation of small deviations at the individual disaccharide level^33^ result in the observed shifts. It is important to note though that the SASA was not used as target data for the parametrization.

**Figure 9:**
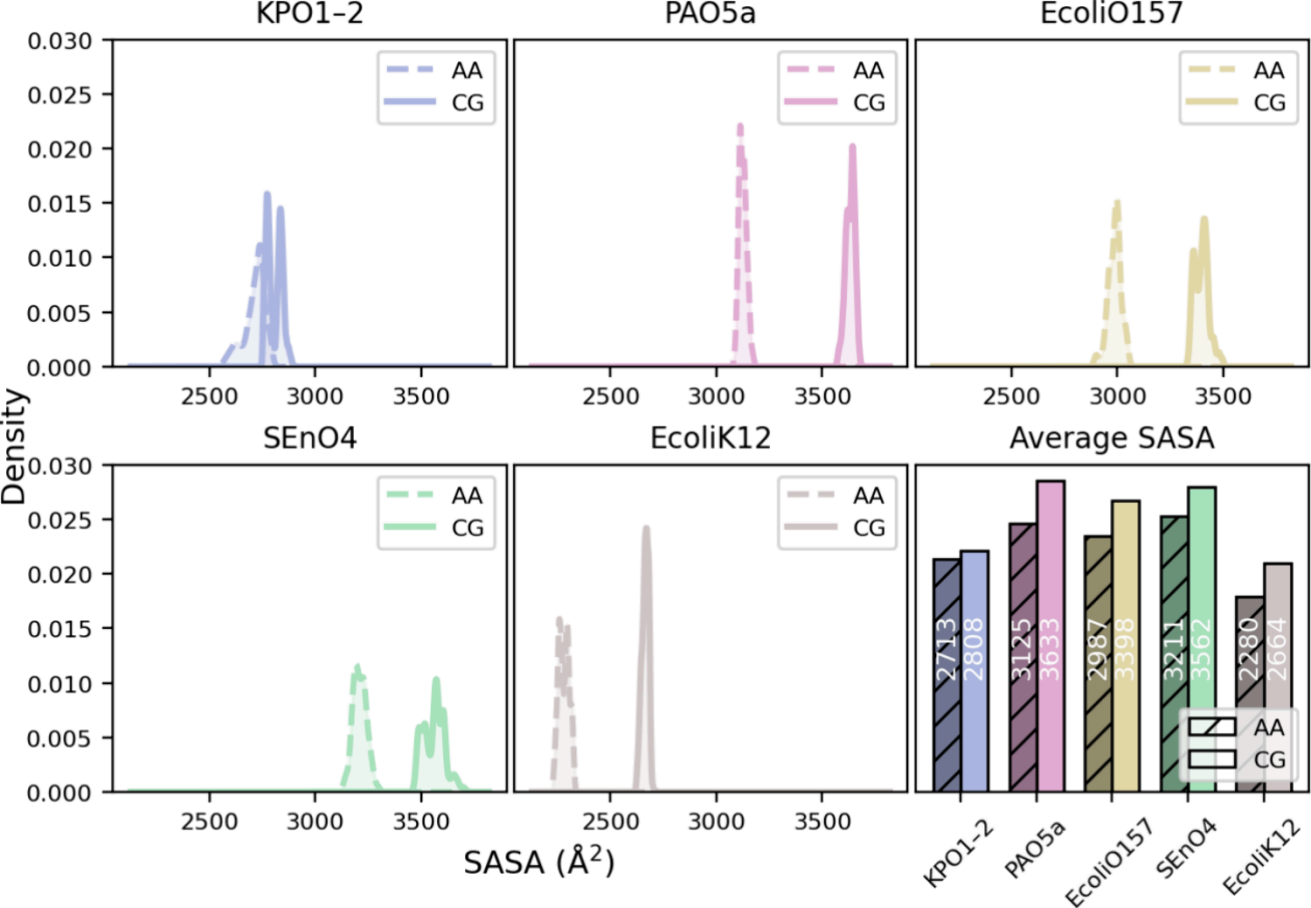
SASA of LPS across all serotypes from CG and AA simulations. For each system, SASA distributions generated from the combined two independent replicas are shown for both AA and CG models, with the corresponding averages displayed in the lower right panel. Corresponding per-replica SASA distributions are provided in the Supporting Information (Figure S12).

#### Mean Square Displacement and Diffusion of LPS

To assess lateral dynamics of LPS, we calculated the mean square displacement (MSD) as a function of the time lag. The analysis was carried out over the last 5 μs of AA simulations and the last 10 μs of CG simulations. The MSD was averaged for window sizes of 2.5 and 5 μs for AA and CG, respectively, similar to previous publications.^49,61–63^ While large-scale lateral exchanges of LPS molecules were not observed, reflecting their intrinsically slower dynamics compared to phospholipids, local motions were still apparent.^64^ By fitting the slope of the MSD (Figure S15) from the last sections of the trajectories (1.25 μs for AA and 2.5 μs for CG), we extracted diffusion coefficients.^49^ In general, the AA diffusion coefficients are in reasonable accord with earlier experimental values, which depending on the particular system, range from 10^–13^ to 10^–9^ cm^2^/s.^65–67^ The CG systems displayed diffusion constants (Table 2) approximately 4 to 25 times faster than their AA counterparts, consistent with the expected acceleration for dynamics in Martini CG models, where diffusion constants 3–6 times larger have been reported.^57,68^

**Table 2:** Diffusion Coefficients (×10^–11^ cm^2^/s) for Each System from AA and CG Simulations with Two Replicas Each

LPS Type	AA Rep1	AA Rep2	CG Rep1	CG Rep2	AA Avg.	CG Avg.
KPO1-2	1.32	1.61	4.52	6.61	1.47	5.57
PAO5a	0.31	0.38	8.68	7.86	0.35	8.27
EcoliO157	0.22	0.53	7.41	7.22	0.38	7.32
SEnO4	1.41	1.64	4.43	4.44	1.53	4.44
EcoliK12	0.51	0.82	4.11	6.24	0.67	5.18

## Discussion

MD simulations provide a bridge between atomic-level descriptions of biologically relevant systems and experimentally accessible information. Given the unique structure and slow dynamics of the OM of Gram-negative bacteria, simulations employing AA force fields are often inadequate for the study of such heterogeneous membrane systems. Moreover, some bacterial resistance mechanisms involve a dynamic alteration of the O-antigenic layer of the OM, which further increases the complexity of these systems. Therefore, the ability to readily alter the O-antigen component of the LPS becomes highly attractive in computational programs addressing Gram-negative bacteria. By reducing the atom count, and also smoothing the potential energy surface, CG simulations offer the computational efficiency necessary to adequately model increasingly large realistic, biologically relevant systems for longer time scales. In this manner simulations have reached spatial and temporal extents unattainable with AA representations.^69^ This is especially beneficial for studying slow LPS dynamics and long-chain O-antigen behavior.

One of the most widely used CG force fields is Martini; however, some deficiencies had been noted, such as overaggregation of proteins^23,24^ and carbohydrates,^25^ leading to an extensive revision and release of the Martini 3 force field, with an expanded coverage of chemical space and revised parametrization strategy.^18^ Moreover, deficiencies in lipid phase behavior have led to a refined mapping and parametrization for lipids, resulting in the recent release of Martini 3-based lipidome parameters.^28^ Although Martini 3 mitigates of many of these earlier deficiencies,^18,70^ detailed studies have shown incorrect partitioning of peptides,^71^ inaccurate TM helix-membrane insertion,^72^ as well as a flip-flop rate that appears too high.^73^

The use of top-down CG force fields such as Martini is not without limitations.^23,56,70,74^ Given the reduction in the degrees of freedom inherent in the Martini CG mapping of AA systems, the entropy is reduced.^18^ Careful studies employing the Martini 2.0 and 3.0 force fields have revealed an inaccurate partitioning of enthalpy/entropy relative to AA results for potentials of mean force in membrane simulations.^56,74^ However, without some temperature dependence built into the model it may be quite difficult to recover the proper thermodynamic partitioning of the enthalpy and entropy. In an effort to overcome some of these limitations, recent modifications of the Martini 3 lipid parameters include the fitting to AA simulations in addition to physical properties.^28^ In this manner a combined top-down and bottom-up approach is taken. It is important to note that even with these modifications, the Martini 3-based simulations produce a barrier for phospholipid flip flop across membrane bilayers that is too low relative to Martini 2.^73^ As such, and much like the continued development of AA force fields,^75^ the Martini CG model continues to evolve.

In this work, we developed and validated a transferable set of Martini 3 parameters for modeling LPS from five medically relevant Gram-negative bacteria. By systematically building LPS models from disaccharide building blocks and validating them against AA simulations, we demonstrated that our CG models reproduce key structural and dynamic features of bacterial OMs.

Conventional CG parametrization of complex glycans typically involves running AA simulations of the entire LPS molecule and mapping the resulting full-molecule trajectories to CG parameters.^31,32^ In contrast, our approach is modular: we parametrize at the disaccharide level and then assemble the O-antigen and core from these building blocks. While this modular concept could in principle be implemented with PyCGTOOL, it would still require more than 60 separate AA disaccharide simulations to extract bonded terms–an extremely tedious task.^32^ In fact a variety of glucose/mannose disaccharides have been parametrized via the PyCGTool package performed against all-atom MD, using a fully automated or semiautomated approach for the bonded terms, including dihedrals.^76^ Noteworthy, as reported by Brandner et al.,^76^ parameter distributions generated employing different AA force fields differ on average by 66%. Such differences suggest a direct comparison can be obscured by the choice of force field. Instead, we used Bartender, which derives bonded parameters directly from small building blocks and can target either AA or QM data.^33^ While AA parameters already exist for the structures studied here, we chose QM targets because Bartender can generate them automatically, eliminating the need for pregenerated AA MD trajectories while still producing accurate bonded terms. This workflow allowed us to efficiently parametrize five LPS systems with diverse O-antigen/core compositions, avoiding the extensive AA simulations and manual refitting steps of conventional methods.

While Martini 3 introduces a broader range of bead types and improved mapping schemes, enhancing the geometric representation of carbohydrates, its default parameters are not necessarily optimal for reproducing some biophysical observables in complex glycan systems. In particular, Brandner et al.^32^ demonstrated that Martini 2, when combined with custom-tuned bonded parameters, can more accurately capture APL and membrane thickness of bacterial OMs. Consistent with these observations, our own results reveal that Martini 3 can exhibit noticeable shifts for some serotypes while performing substantially better for others. For KPO1-2 and SEnO4, the APL distributions are shifted by roughly 20 Å^2^ relative to their AA counterparts, whereas PAO5a, EcoliO157, and EcoliK12 show much closer agreement between resolutions. A similar trend is observed for membrane thickness: SEnO4, EcoliO157, and EcoliK12 are reproduced within approximately 5% of the AA values, comparable to the Martini 2 performance reported by Brandner et al.,^32^ whereas PAO5a and KPO1-2 exhibit upward shifts of up to 13%, consistent with the deviations reported for Martini 3 in the same study. While Martini 3 offers general improvements in chemical diversity and mapping fidelity, the agreement with AA benchmarks depends on both model-specific features and parametrization choices for a given system. The differences observed here are modest and likely reflect a combination of these factors rather than a fundamental performance advantage of one Martini version over the other.

Because our approach is modular and efficient, parametrizing additional LPS serotypes is now straightforward, requiring only the parametrization of the novel disaccharide units. This is particularly important given that even a single bacterial species can express dozens of distinct serotypes, each differing in O-antigen composition and eliciting different antibody responses. Across multiple serotypes and simulation replicas, our CG models capture essential biophysical properties of LPS, including O-antigen tilt, end-to-end extension, membrane thickness, and area per LPS, giving close agreement with AA results. These structural features shape the conformational landscape of LPS, which influences antigen accessibility, immune evasion, and interactions with antimicrobial peptides. Notably, the density distribution of charged groups (phosphate and carboxylate) and divalent cations showed strong consistency between CG and AA simulations. This finding supports the validity of Martini 3 parameters in preserving these aspects of the electrostatic organization of the OM, which plays a critical role in maintaining barrier integrity and coordinating ion-mediated stabilization of LPS layers.

Some small deviations were observed in local structural metrics, such as slight differences in density peak positions or tilt angle distributions. These may arise from differences in bead-mapping schemes or in bonded-parameter approximations. Although further refinement of bonded parameters (e.g., modification of glycosidic angle terms or inclusion of torsional potentials) could potentially reduce specific deviations in observables such as APL or membrane thickness, our primary objective was to preserve a minimal and transferable building-block framework. In this approach, each disaccharide constitutes the fundamental parametrized unit, and no linkage-specific reoptimization was performed at the level of the full polysaccharide. Membrane properties such as area per LPS, bilayer thickness, and O-antigen extension are strongly coupled; tuning one structural metric in isolation can induce compensatory shifts in others. Therefore, aggressive system-specific refinement risks overfitting individual serotypes and compromising transferability across chemically diverse LPS structures. The modest systematic shifts observed here reflect this deliberate design choice rather than an inability to further tune individual systems. Our approach opens the door for future studies on immune recognition, antibiotic permeability, OM vesicle dynamics, and multicomponent membrane-protein systems.

Beyond conventional structural and dynamical observables, we also examined the topological organization of the O–antigen layer by quantifying pairwise entanglement between LPS molecules using the Gaussian Linking Number (GLN). The GLN provides a measure of the average winding of two polymer-like chains and has previously been used to characterize entanglement in biopolymers and macromolecular assemblies.^53,77^ Across all five LPS systems, the GLN distributions obtained from CG and AA simulations showed remarkably similar shapes and magnitudes, indicating that the Martini 3 representation faithfully preserves the mesoscale topological organization of the O-antigen layer. In all simulations, GLN values remained low, consistent with the expectation that O–antigen chains are highly flexible and predominantly fluctuate within the plane of the membrane rather than forming persistent topological links (GLN > 1). This behavior reflects chains that approach or transiently overlap in projection but do not topologically wrap around one another. The close agreement between resolutions further suggests that Martini 3 accurately captures the relative spacing, orientational freedom, and steric interactions that collectively define the entanglement landscape of the LPS layer. Importantly, because entanglement can influence chain mobility the ability of the CG model to reproduce this feature strengthens confidence in its use for studying large-scale collective dynamics of bacterial OMs.

The role of Ca^2+^ in the structure and modulating LPS dynamics has been previously highlighted in both experimental and simulation studies.^60^ Schenck et al.^58^ have shown that divalent cations predominately occupy the region between the inner core and lipid A, while Clifton et al.^59^ have demonstrated that removing calcium ions leads to significant disruption of the OM, with increased mixing of inner leaflet phospholipids and outer leaflet LPS. Moreover, Ca^2+^ binding is known to compact and rigidify the LPS-containing membrane, as shown by Rice et al.^78^ This is consistent with observations from Kim et al.,^36^ who reported significantly reduced lateral mobility of LPS when divalent Ca^2+^ ions were used instead of monovalent cations. These findings support the interpretation that Ca^2+^ not only stabilizes LPS packing by neutralizing negative charges and promoting tighter molecular organization but also reduces mobility, reinforcing the importance of accurate electrostatics in CG models.

In addition to structural metrics, we also evaluated the dynamic behavior of LPS using mean square displacement (MSD) analysis. As LPS diffuses much more slowly than phospholipids,^20,65,66^ direct lateral exchange of LPS molecules was not anticipated, yet the MSD curves demonstrated detectable dynamics. As expected, the CG models displayed diffusion rates approximately 4 to 25 times faster than the atomistic ones, consistent with the acceleration typically associated with CG resolution. Importantly, despite this speed-up, it still remains about 1000× slower than the diffusion of phospholipids reported in previous CG simulations.^28,31,64,79^ The diffusion coefficients presented here are lower than values obtained in earlier CG LPS studies,^20,80^ which reported diffusion on the order of 10^–10^-10^–8^ cm^2^/s. Aside from the use of Martini 2 parameters, which produce slightly larger diffusion constants,^28^ these earlier studies considered LPS species with reduced acylation and/or without extended O-antigen chains. In contrast, the systems investigated here contain hexa- or hepta-acylated lipid A (six or seven acyl chains) and two O-antigen repeats, which enhance membrane packing and reduce lateral mobility. Therefore, the slower diffusion observed in the present work most likely reflects differences in molecular architecture and membrane composition rather than limitations of the CG representation.

## Conclusions

Given the unique structure of the OM of Gram-negative bacteria and the central role the outer leaflet plays in bacterial virulence, survivability, and antigenic residence, efficient computational approaches are necessary. In particular the presence of the O-antigen layer, a flexible polysaccharide of highly variable composition, makes sampling via AA-MD problematic. Gram-negative bacteria are known to evade host immune responses by alteration in composition and/or length of the O-antigen component of the OM; therefore a computational pipeline that allows for systematic and rapid reparametrization is necessary. Moreover, the use of CG approaches is highly attractive due to their computational efficiency, while retaining sufficient underlying details of the molecular interactions. The recent update of the Martini 3 force field and the use of semiautomatic parametrization tools such as Bartender proved an attractive method to rapidly parametrize LPS from several bacterial pathogens with highly diverse O-antigen composition in a building-block manner, making this approach sufficiently agile for the construction and simulation of model bacterial OMs. Here we have shown that this approach can readily provide the necessary parameters for multiple bacterial species of alternate O-antigen composition at the level of accuracy obtainable with the current class of Martini CG models. Thus, our building-block approach provides a promising avenue for modeling the OMs of medically relevant Gram-negative bacteria.

Beyond providing a validated parameter set, this work establishes a reproducible and extensible framework for constructing serotype-specific LPS models from modular disaccharide building blocks. By requiring parametrization only of new disaccharide units, the approach enables systematic variation of O-antigen length and composition within a consistent Martini 3 representation, without the need for additional full-length AA reference simulations. This makes it feasible to directly probe how defined glycan features influence membrane packing, ion organization, and mesoscale properties of the OM at time scales inaccessible to atomistic models. In this way, the present framework provides a practical foundation for comparative studies of Gram-negative outer membranes across serotypes and environmental conditions.

## Supplementary Material



## Data Availability

All data supporting the findings of this study, including the final conformations from AA and CG simulations, developed parameter files, and the script for combining disaccharide parameters, are openly available on Zenodo at 10.5281/zenodo.18009953.

## Notes

Additional figures and analysis, including full LPS composition, parameter fitting data, and simulation system properties and measurements (PDF)
