# Supplementary material for: Martini 3 as a Transferable Force Field for Lipopolysaccharide Parametrization
Source: J Phys Chem B. 2026 Mar 12;130(12):3369–83. doi: 10.1021/acs.jpcb.6c00050 (PMC13034417; doi:10.1021/acs.jpcb.6c00050)
Supplement: Supplementary file 1 [file jp6c00050_si_001.pdf]

Supporting Information: Martini 3 as a Transferable Force Field for Lipopolysaccharide Parameterization

Gvantsa Gutishvili,† Diane L. Lynch,† and James C. Gumbart*,†,‡

†*School of Physics, Georgia Institute of Technology, Atlanta, Georgia, 30332, USA*

‡*School of Chemistry & Biochemistry, Georgia Institute of Technology, Atlanta, Georgia, 30332, USA*

E-mail: gumbart@physics.gatech.edu

LPS Composition

Table S1: LPS Composition in CHARMM-GUI notation

System	Lipid A	Core Oligosaccharide	O-Antigen
EcoliK12[1]	Type 1	K12: -(6-2)αDKdo-[(4-2)αDKdo]-(5-1)αLDHep-(3-1)αLDHep-[(7-1)αLDHep]-(3-1)αDGlc-(3-1)αDGlc-[(6-1)αDGal]-(2-1)αDGlc-(6-1)αLDHep	-
EcoliO157[1]	Type 1	R3: -(6-2)αDKdo-[(4-2)αDKdo]-(5-1)αLDHep-(3-1)αLDHep -[(7-1)αLDHep]-(3-1)αDGlc- (3-1)αDGal-[(3-1)αDGlcNAc] -(2-1)αDGlc-[(2-1)αDGlc]-(4-)	O157: (-1)αDGalNAc-(3-1)βDGlc-(4-1)αLFuc-(3-1)αDRha(2-)
SEnO4[2]	Type1	-(6-2)αDKdo-[(4-2)αDKdo]-(5-1)αLDHep-(3-1)αLDHep-[(7-1)αLDHep]-(3-1)αDGlc-[(6-1)αDGal] -(3-1)αDGal-(2-1)αDGlc-[(2-1)αDGlcNAc]-(4-)	O4-1: (-1)αDGal-[(4-1)αDGlc]-(3-1)αLRha-(4-1)αDMan-[(3-1)αDAbe]
KPO1-2[3]	Type 2	A: -(6-2)αDKdo-[(4-2)αDKdo]-(5-1)αLDHep-[(4-1)βDGlc-(6-1)βDGalA]-(3-1)αLDHep-[(7-1)αLDHep-(7-1)βDGalA]-(3-1)αDGalA-(4-1) βDGlcN-(6-2)αDKdo-[(4-1)αLDHep](5-)	O1-2: (-1)αDGal-(3-1)βDGal-(3-)
PAO5a[4]	Type 1	2:-(6-2)-αDKdo-[(4-2)αDKdo]-(5-1)αLDHep-(3-1)αLDHep-(3-1)αDGal-[(4-1)αDGlc]-(3-1)βDGlc-[(6-1)αDGlc]-(3-1)αLRha-(3-)	O5a: (-1)αDFuc-(3-1)βDManNAc-(4-1)βDManNAc-(4-)

[1] *Escherichia coli*, [2] *Salmonella enterica*, [3] *Klebsiella pneumoniae*, [4] *Pseudomonas aeruginosa*

Table S2: List of disaccharides used in the Martini 3 LPS parameterization. Disaccharides marked with the same number of asterisks (*, **, *) indicate identical building blocks reused across different serotypes.**

ID	Serotype	Disaccharide	ID	Serotype	Disaccharide
1	EcoliK12	αDKdo(4–2) αDKdo**	33	SEnO4	βDGal(4–1) αDGlc
2	EcoliK12	αDKdo(5–1) αLDHep*	34	SEnO4	βDGal(3–1) αLRha
3	EcoliK12	αLDHep(3–1) αLDHep	35	SEnO4	αLRha(4–1) αDMan
4	EcoliK12	αLDHep(7–1) αLDHep	36	SEnO4	αDMan(3–1) αDAbe
5	EcoliK12	αLDHep(3–1) αDGlc***	37	SEnO4	αDMan(2–1) αDGal
6	EcoliK12	αDGlc(3–1) αDGlc	38	KPO1–2	αDKdo(4–2) αDKdo**
7	EcoliK12	αDGlc(6–1) αDGal	39	KPO1–2	αDKdo(5–1) αLDHep*
8	EcoliK12	αDGlc(2–1) αDGlc	40	KPO1–2	αLDHep(4–1) βDGlc
9	EcoliK12	αDGlc(6–1) αLDHep	41	KPO1–2	βDGlc(6–1) βDGalA
10	EcoliO157	αDKdo(4–2) αDKdo**	42	KPO1–2	αLDHep(3–1) αLDHep
11	EcoliO157	αDKdo(5–1) αLDHep*	43	KPO1–2	αLDHep(7–1) αLDHep
12	EcoliO157	αLDHep(3–1) αLDHep	44	KPO1–2	αLDHep(7–1) βDGalA
13	EcoliO157	αLDHep(7–1) αLDHep	45	KPO1–2	αLDHep(3–1) αDGalA
14	EcoliO157	αLDHep(3–1) αDGlc***	46	KPO1–2	αDGalA(4–1) βDGlcN
15	EcoliO157	αDGlc(3–1) αDGal	47	KPO1–2	βDGlcN(6–2) αDKdo
16	EcoliO157	αDGal(3–1) αDGlcNAc	48	KPO1–2	αDKdo(4–1) αLDHep
17	EcoliO157	αDGal(2–1) αDGlc	49	KPO1–2	αDKdo(5–1) βDGal
18	EcoliO157	αDGlc(2–1) αDGlc	50	KPO1–2	βDGal(3–1) βDGal
19	EcoliO157	βDGlc(4–1) βDGalNAc	51	KPO1–2	βDGal(3–1) αDGal
20	EcoliO157	βDGalNAc(3–1) βDGlc	52	PAO5a	αDKdo(4–2) αDKdo**
21	EcoliO157	βDGlc(4–1) αLFuc	53	PAO5a	αDKdo(5–1) αLDHep
22	EcoliO157	αLFuc(3–1) αDRha	54	PAO5a	αLDHep(3–1) αLDHep
23	EcoliO157	αDRha(3–1) αDGalNAc	55	PAO5a	αLDHep(3–1) αDGal
24	SEnO4	αDKdo(4–2) αDKdo**	56	PAO5a	αDGal(4–1) αDGlc
25	SEnO4	αDKdo(5–1) αLDHep	57	PAO5a	αDGal(3–1) βDGlc
26	SEnO4	αLDHep(3–1) αLDHep	58	PAO5a	βDGlc(6–1) αDGlc
27	SEnO4	αLDHep(7–1) αLDHep	59	PAO5a	βDGlc(3–1) αLRha
28	SEnO4	αLDHep(3–1) αDGlc	60	PAO5a	αLRha(3–1) βDFuc
29	SEnO4	αDGal(6–1) αDGal	61	PAO5a	βDFuc(3–1) βDManNAc
30	SEnO4	αLDHep(3–1) αDGal	62	PAO5a	βDManNAc(4–1) βDManNAc
31	SEnO4	αDGal(3–1) αDGlc	63	PAO5a	βDManNAc(4–1) αDFuc
32	SEnO4	αDGlc(4–1) βDGal	64	SEnO4	αDGlc(2–1) αDGlcNAc

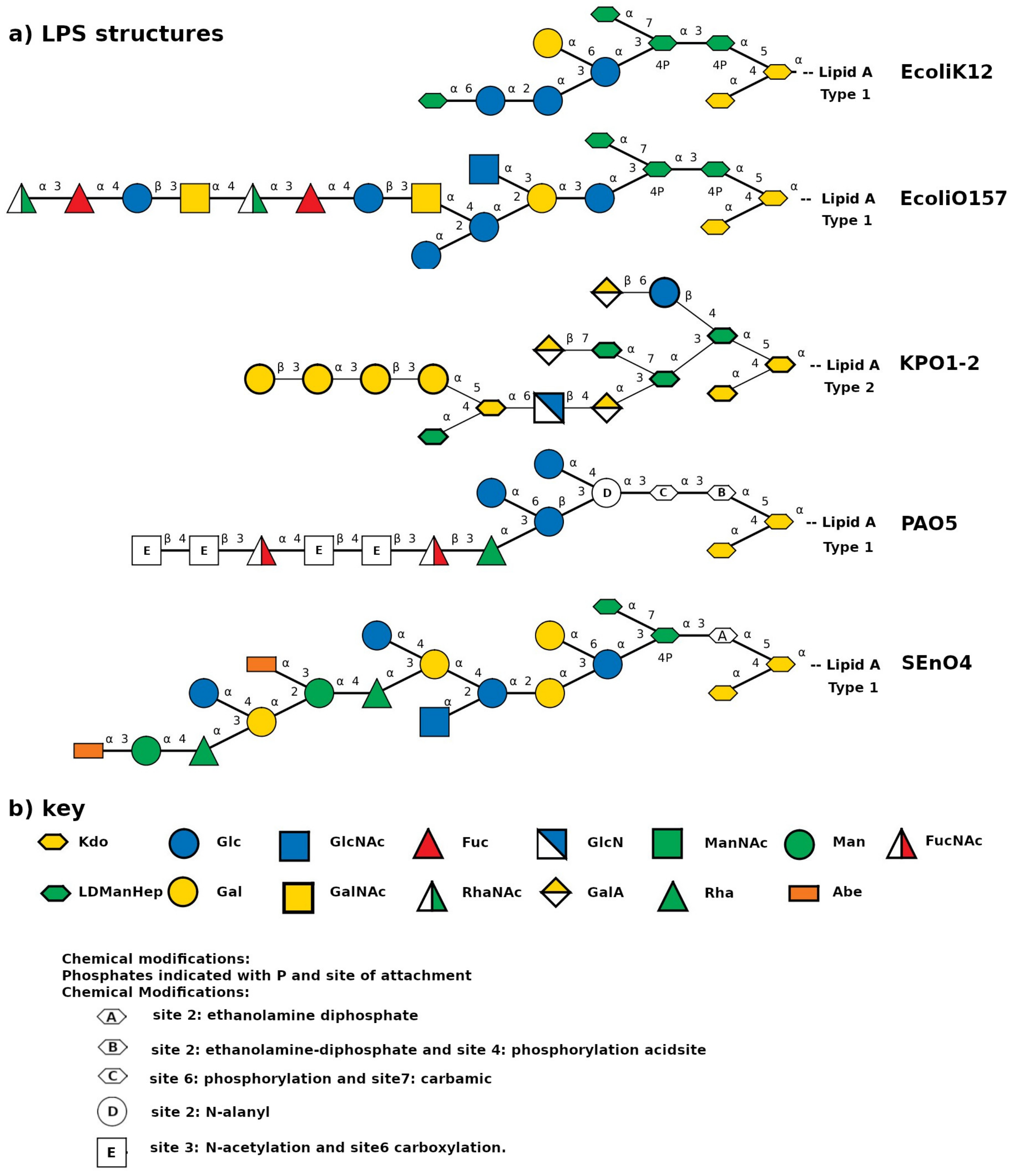

Figure S1: Composition of LPS using symbol nomenclature for glycans (SNFG):[S1] a) LPS of each bacterial system. b) key for symbols, including the chemical modification, if present, for each saccharide.

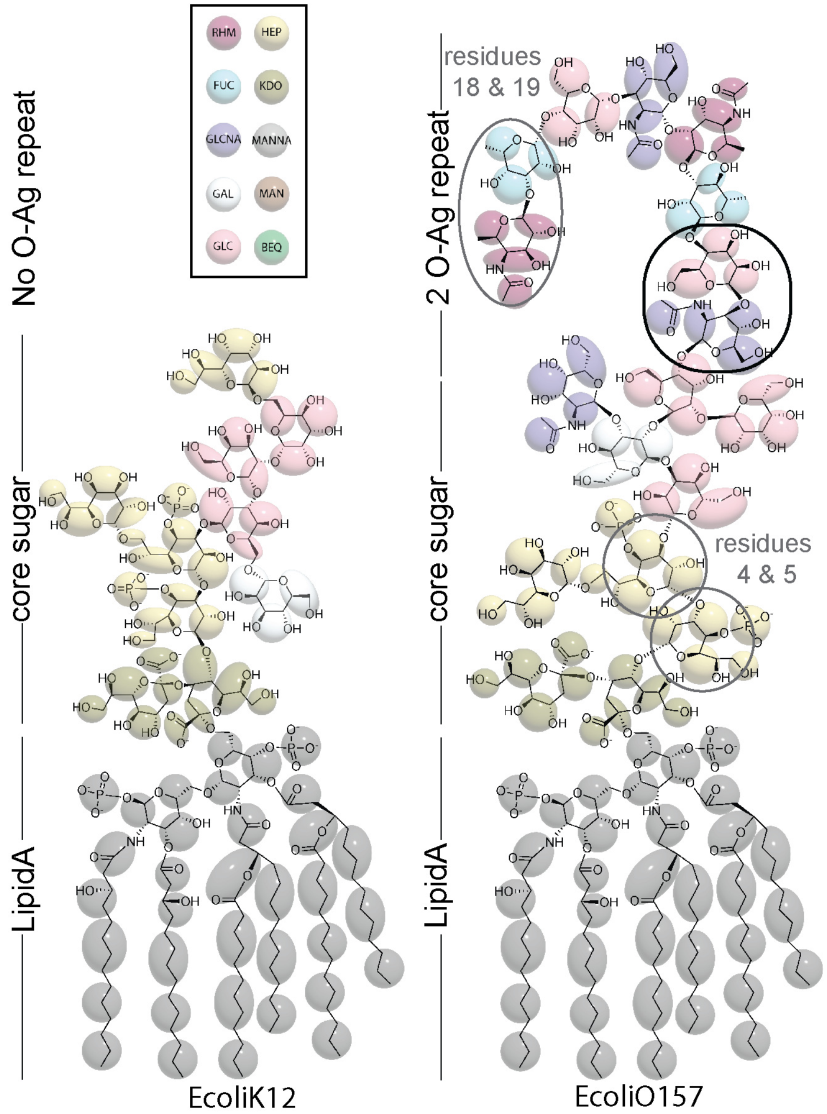

Figure S2: Structures of *E. coli* K12 and O157 lipopolysaccharides. The schematic highlights the details in core oligosaccharide and O-antigen composition in these two serotypes.[S2,S3] Shaded areas depict Martini 3 beads with the grey beads representing the lipid A (ECLIPA), and other colors: red, yellow, cyan, olive, purple, light gray, white, brown, pink, green are Rhamnose (RHM), Heptose (HEP), Fucose (FUC), 3-deoxy-D-manno-oct-2-ulosonic acid (KDO), N-acetyl-D-glucosamine (GLCNA), N-acetyl-D-mannosamine (MANNA), Galactose (GAL), Mannose (MAN), Glucose (GLC), Abequose (BEQ). The β-glucose (1$\rightarrow$3) β-N-acetyl galactosamine pair is outlined using a black circle.

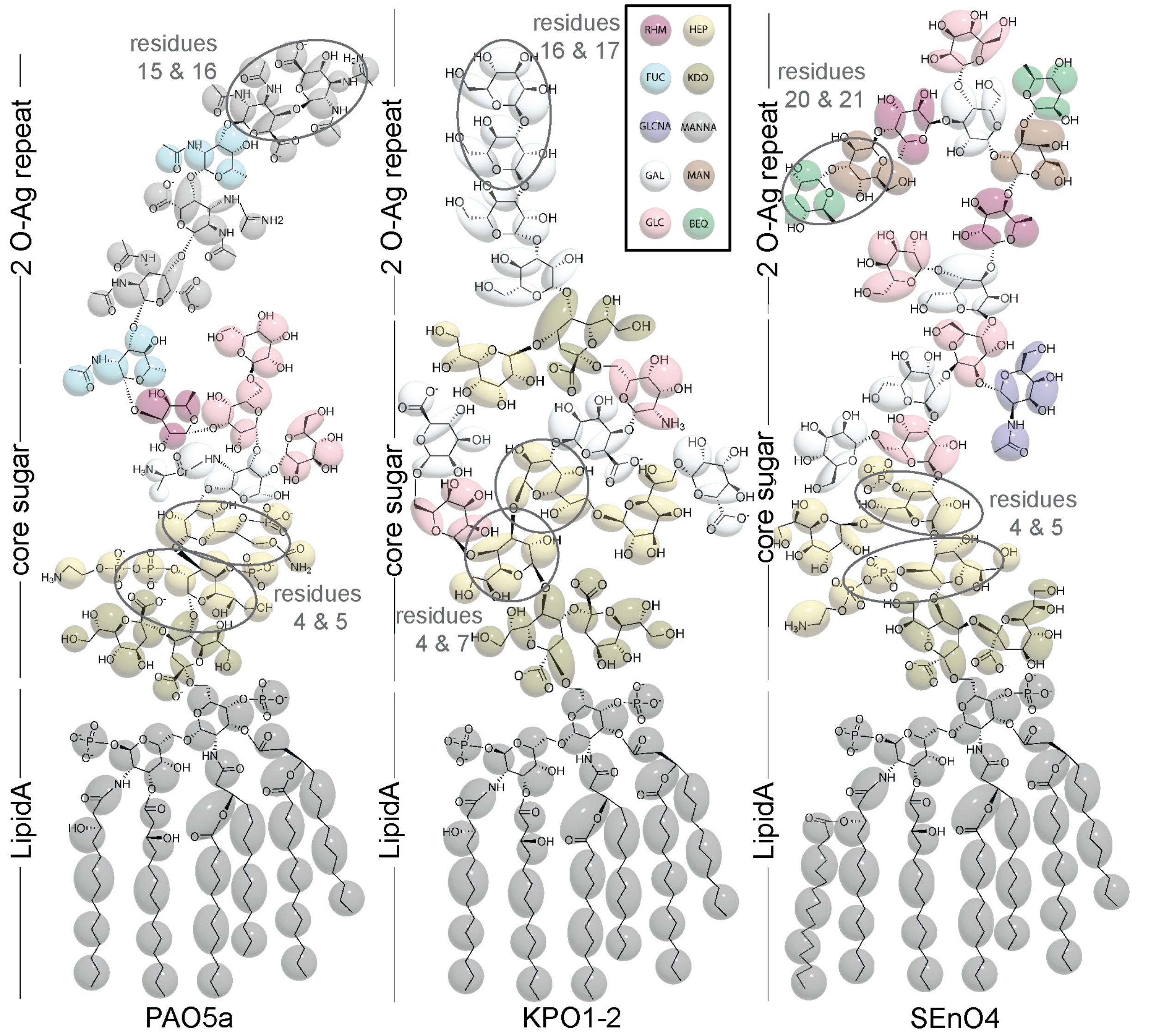

Figure S3: Structures of *P. aeruginosa* O5 (PAO5), *K. pneumoniae* O1-2 (KPO1-2), and *S. enterica* O4 lipopolysaccharides (LPS). The schematics illustrate the distinct core oligosaccharide and O-antigen compositions characteristic of each serotype.[S4–S7] Shaded areas depict Martini 3 beads with the grey beads representing the lipid A (ECLIPA), and other colors: red, yellow, cyan, olive, purple, light gray, white, brown, pink, green are Rhamnose (RHM), Heptose (HEP), Fucose (FUC), 3-deoxy-D-manno-oct-2-ulosonic acid (KDO), N-acetyl-D-glucosamine (GLCNA), N-acetyl-D-mannosamine (MANNA), Galactose (GAL), Mannose (MAN), Glucose (GLC), Abequose (BEQ).

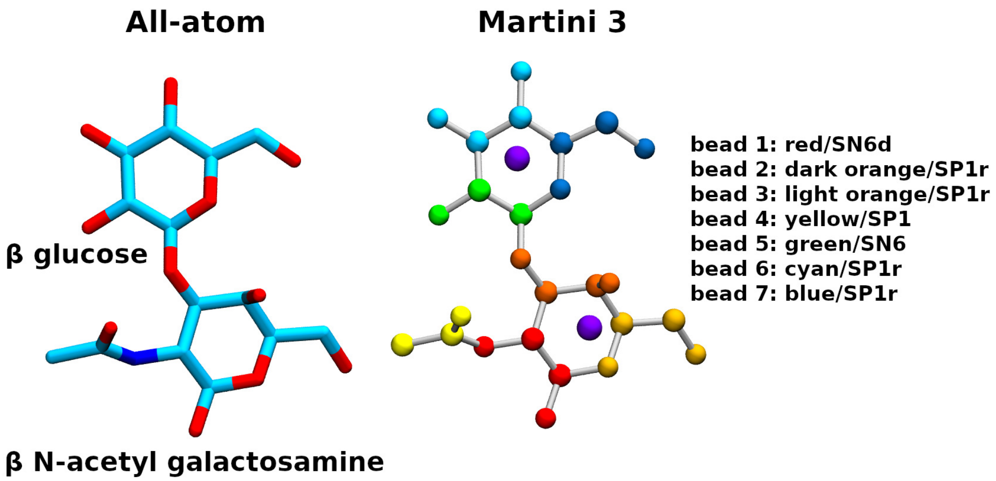

Figure S4: AA to CG mapping of the β-glucose (1→3) N-acetyl galactosamine - disaccharide component of *E. coli* O157. This is the pair of sugars indicated in Fig. 1 of the main text. The carbon/oxygen/nitrogen atoms are rendered cyan/red/blue respectively in the all-atom panel on the left. Hydrogen atoms are not rendered for clarity. In the Martini 3 panel the atoms comprising each of the seven beads is colored by bead number. The purple spheres are virtual sites included in the sugar rings.

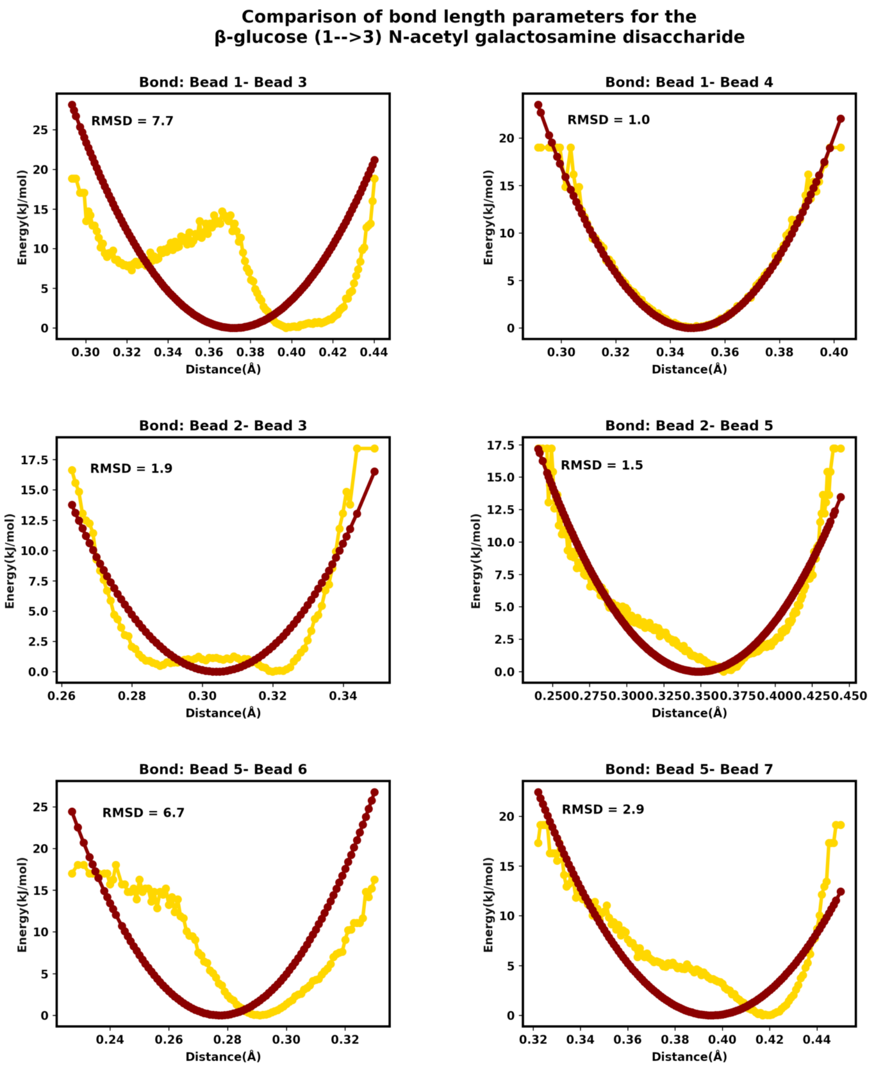

Figure S5: Comparing the CG fit to the QM trajectory for the inter-bead bonds for β-glucose (1→3) N-acetyl galactosamine - disaccharide component of *E. coli* O157. Here the fit/QM trajectory data is rendered in darkred/gold respectively. The RMSD between the Bartender fit and the QM trajectory energy is reported in each panel. Note, a simple harmonic can not fit the double-well structure for the Bead1-Bead3 interaction. Overall the shifts in the bead distances between the Bartender optimized parameters and the QM values are all within the reported RMSE for bond distances of 0.2 Å.[S8]

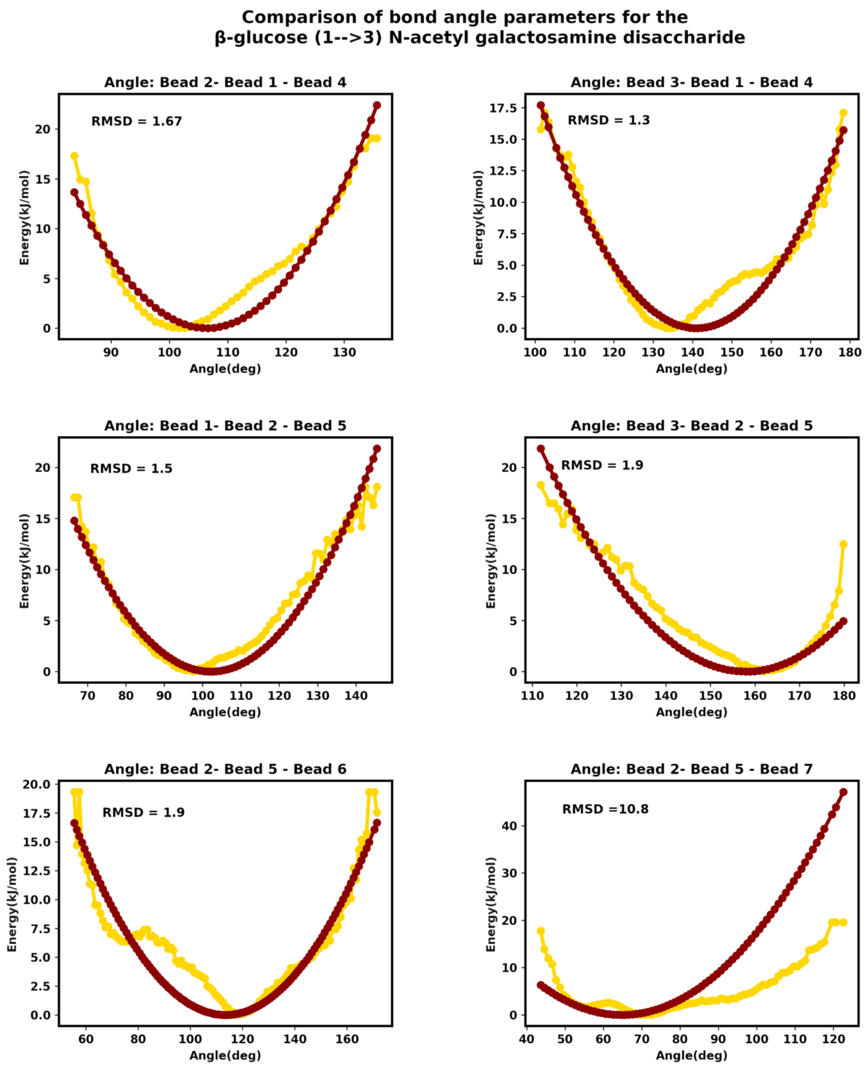

Figure S6: Comparing the CG fit to the QM trajectory for the inter-bead angles for β-glucose (1→3) N-acetyl galactosamine - disaccharide component of *E. coli* O157. Here the fit/QM trajectory data is rendered in darkred/gold respectively. The RMSD between the Bartender fit and the QM trajectory energy is reported in each panel. Note that in the case of the poor fit for Bead2-Bead5-Bead7, manual adjustment of the angle parameter was performed.

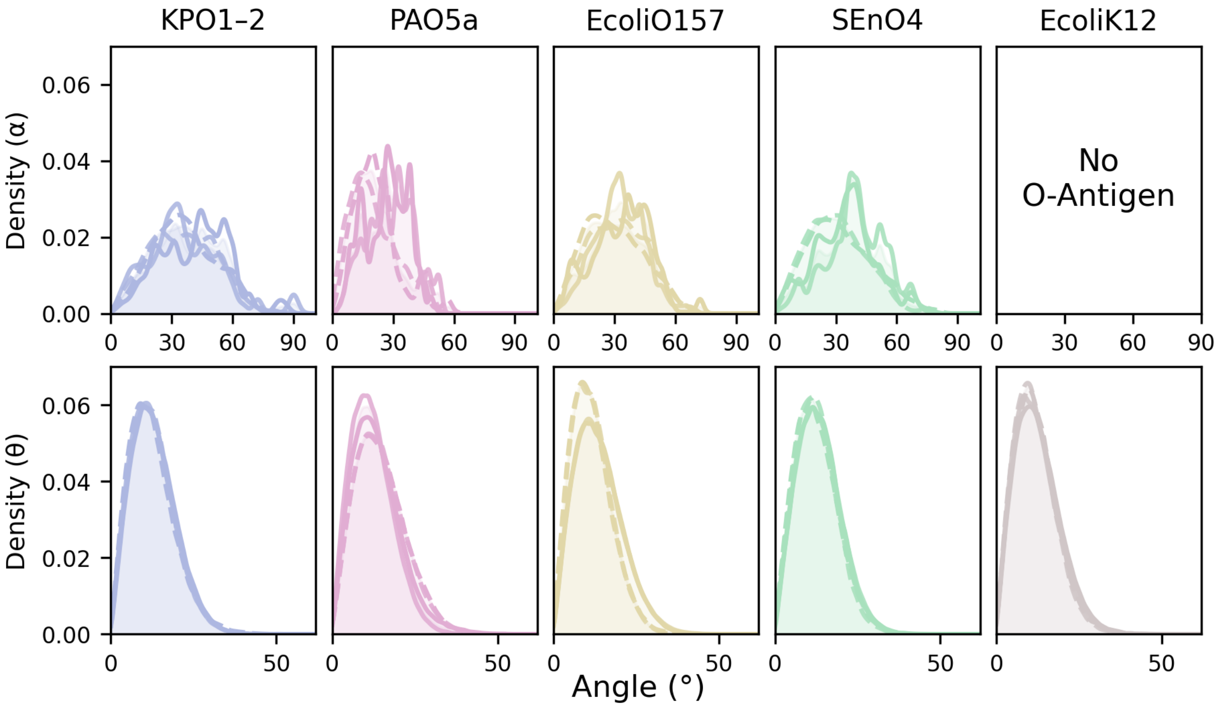

Figure S7: Tilt angle distributions of the polysaccharide (α) and lipid A tail (θ) regions. Distributions are shown for AA and CG simulations, including two independent replicas each. Definitions of the α and θ tilt angles are provided in the text.

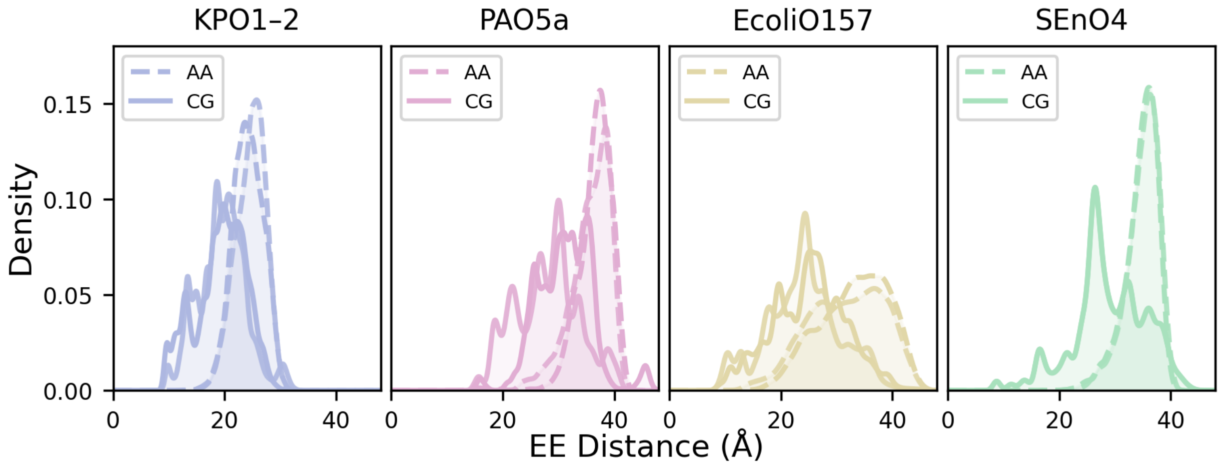

Figure S8: End-to-end distance distributions of the polysaccharide region of LPS molecules for each serotype, comparing AA and CG simulations. For each system and representation, density distributions from two independent replicas are shown.

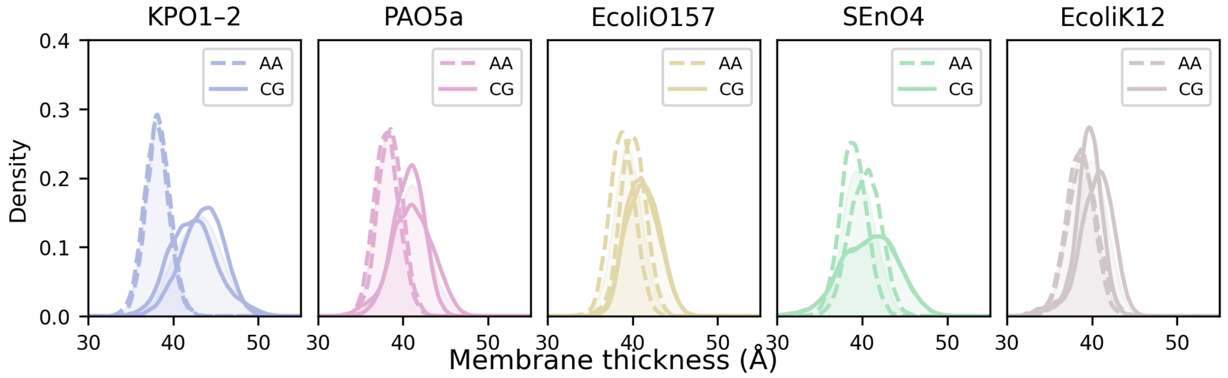

Figure S9: Per-replica membrane thickness distributions for LPS-containing bilayers in CG and AA simulations. For each serotype, membrane thickness probability distributions are shown separately for both AA replicas and both CG replicas, allowing assessment of replica variability and consistency across resolutions. The distributions exhibit similar qualitative shapes between replicas, with CG models systematically producing slightly larger membrane thicknesses relative to AA. These per-replica data complement the averaged thickness distributions shown in the main text (Fig. 5), providing a detailed view of reproducibility across independent simulations.

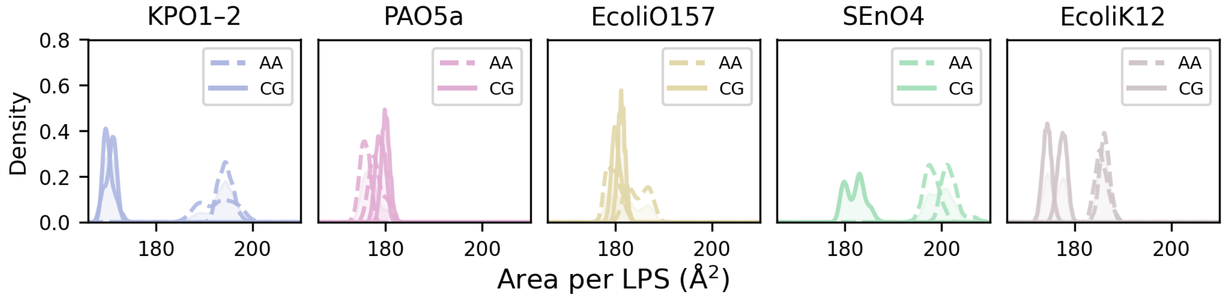

Figure S10: APL distributions from two independent replicas of AA and CG simulations for each LPS serotype. Individual AA (dashed) and CG (solid) replicas are shown separately, allowing assessment of replica-to-replica variability and confirming the reproducibility of area-per-LPS measurements across simulations. These data complement the averaged APL distributions and summary statistics presented in the main text (Fig. 6).

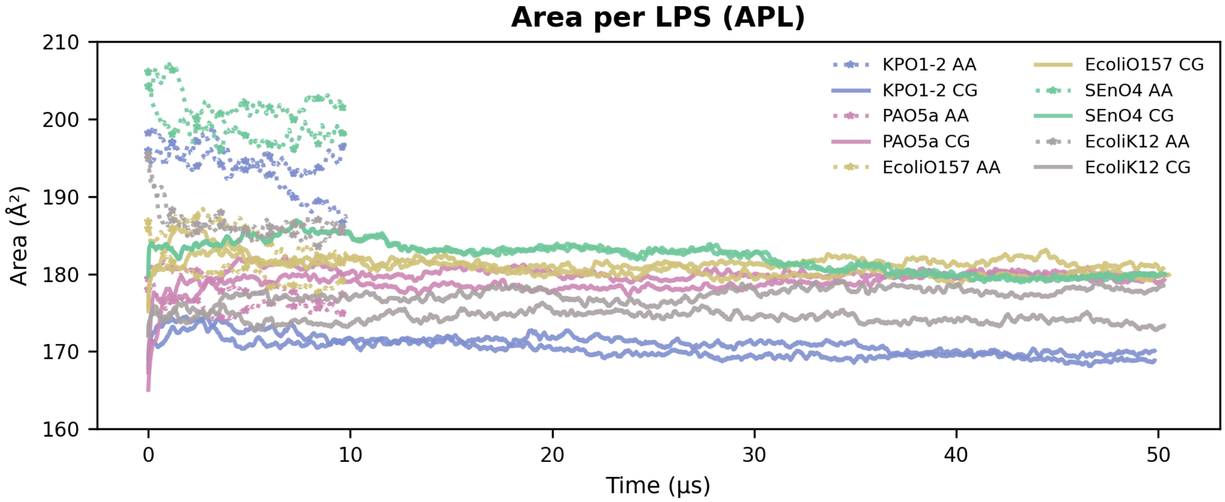

Figure S11: APL for all serotypes from CG and AA simulations. APL values are plotted for each system and replicate, showing consistent trends across serotypes. As expected, CG models generally produce lower APL values than AA models, in agreement with earlier studies reporting tighter packing in CG membranes.[S9,S10] A similar reduction in APL is also observed in AA simulations with hydrogen mass repartitioning (HMR),[S11] further supporting this effect.

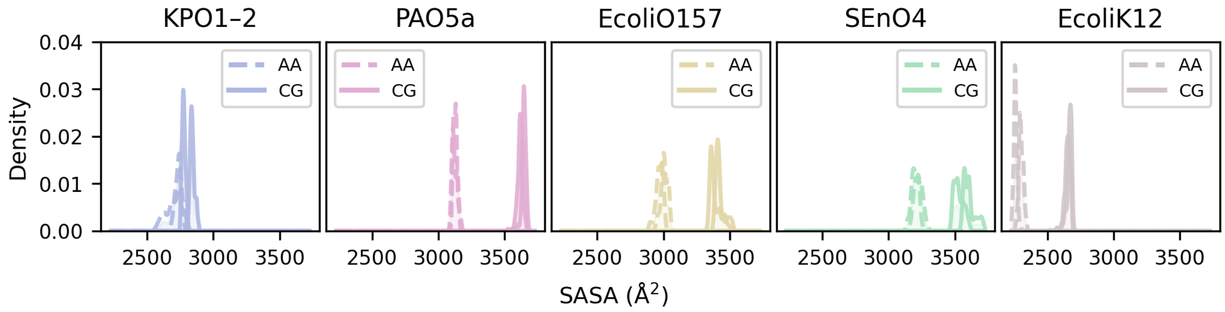

Figure S12: SASA distributions from two independent replicas of AA and CG simulations for each LPS serotype. Individual AA (dashed) and CG (solid) replicas are shown separately, illustrating replica-to-replica variability and confirming the reproducibility of SASA across simulations. These data complement the combined-replica SASA distributions presented in the main text (Fig. 9).

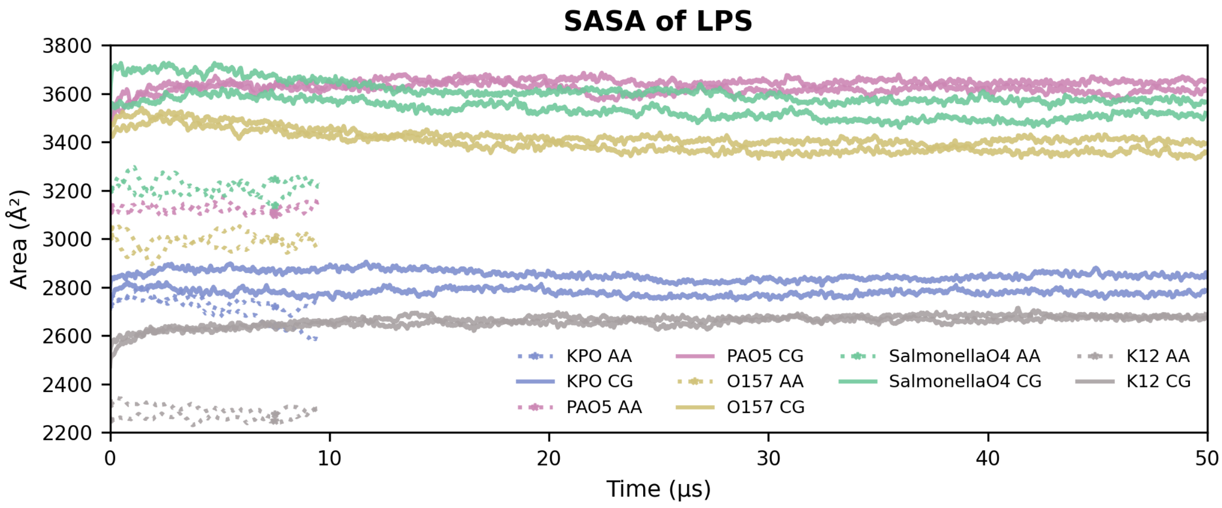

Figure S13: SASA of LPS as a function of simulation time for all serotypes, comparing CG (solid lines) and AA (dashed lines) simulations. For each system, two independent CG and AA replicates are shown. CG simulations yield systematically higher SASA values than AA.

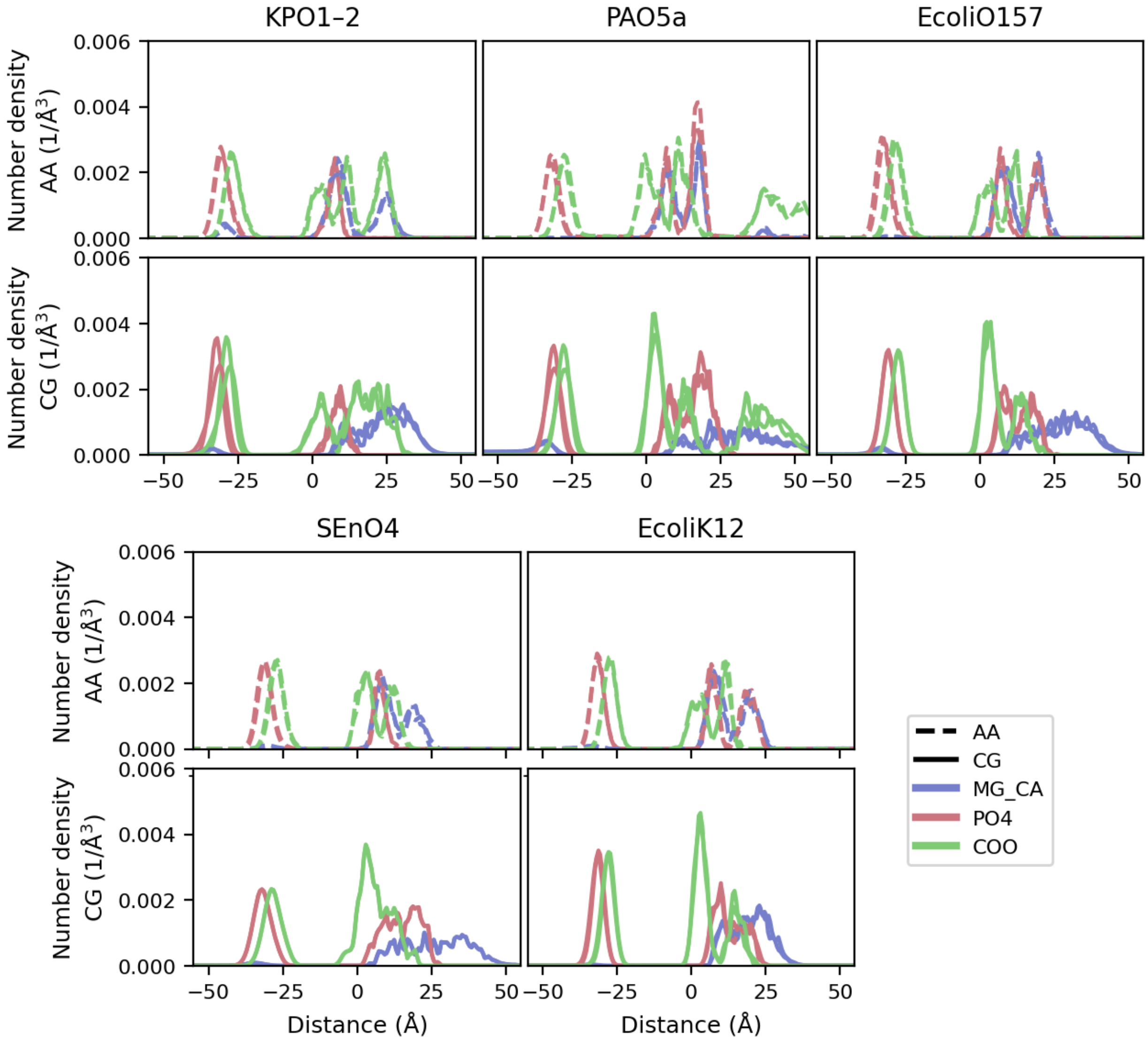

Figure S14: Number density distributions were calculated for charged groups such as phosphate ($PO_4^{2}-$, red) and carboxylate (COO^{-}, green), as well as for divalent cations (Ca^{2+} and Mg^{2+}, blue), along the membrane normal.

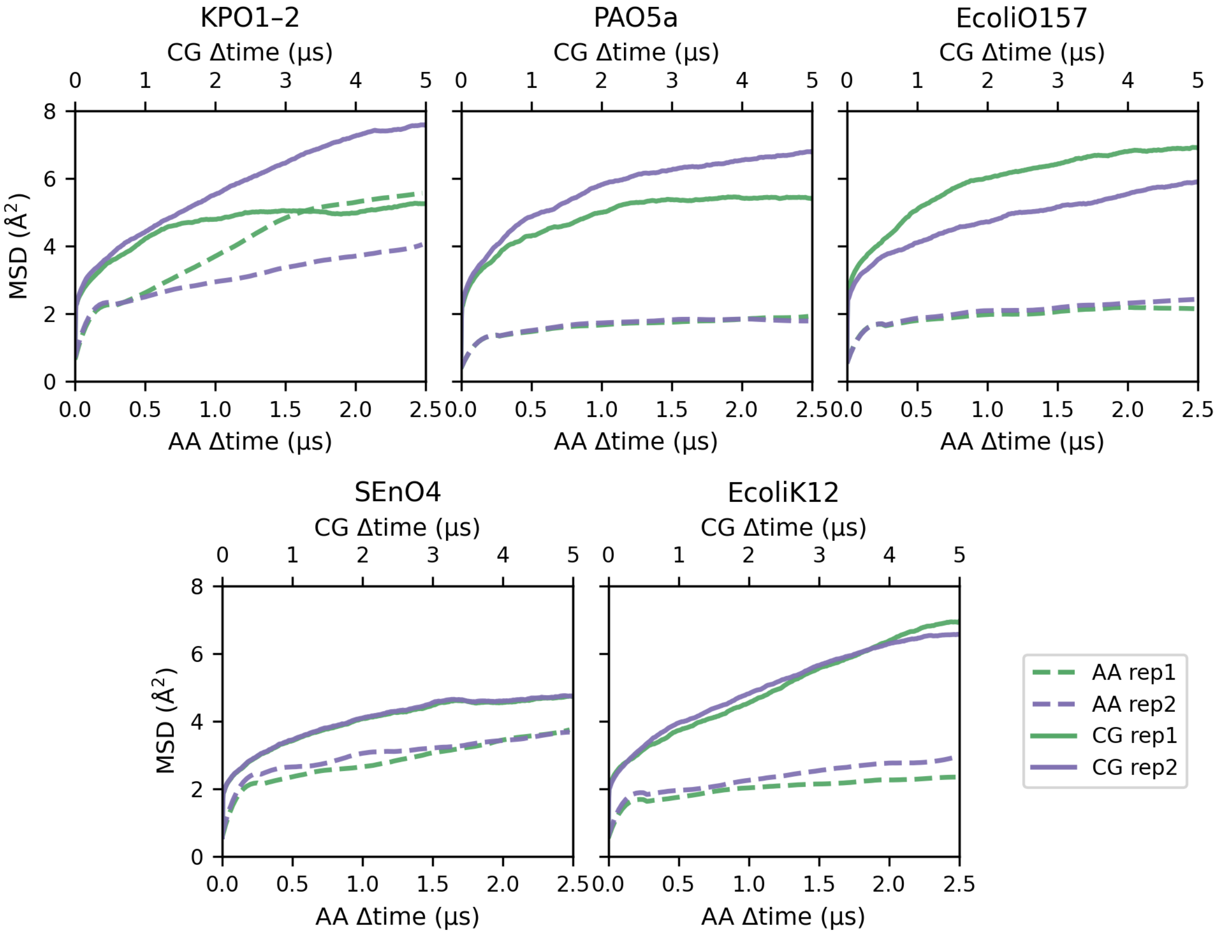

Figure S15: Mean square displacement (MSD) of LPS in AA and CG simulations. MSD was computed from the last 5 μs of AA trajectories and the last 10 μs of CG trajectories. Diffusion coefficients were obtained from the slope of the MSD at last part of trajectories (1.25 μs for AA and 2.5 μs for CG).

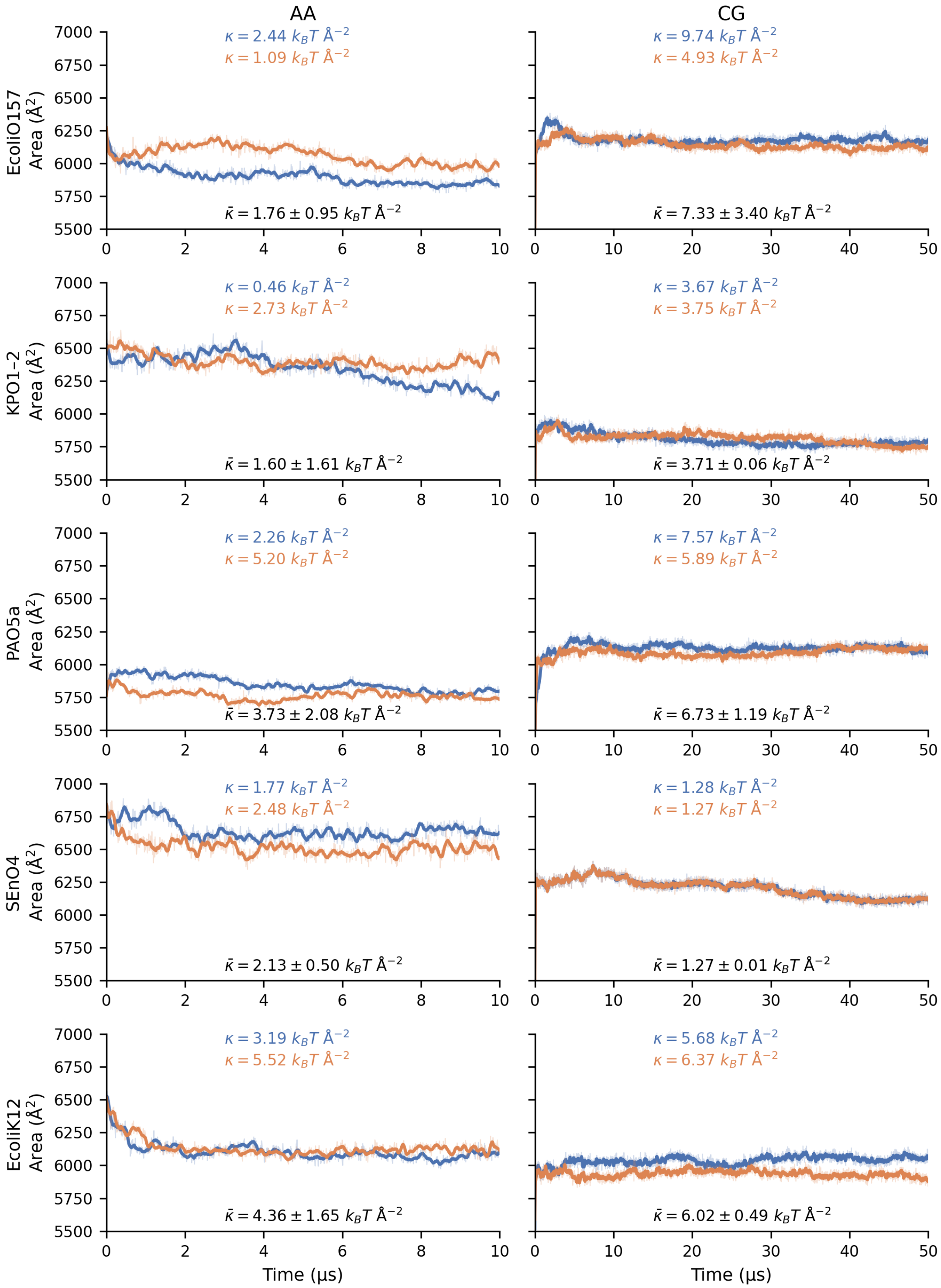

Figure S16: Membrane area over time and membrane compressibility for CG and AA simulations. Calculations were performed following the approach of Balusek et al.[S11]

References

(S1) Cheng, K.; Zhou, Y.; Neelamegham, S. DrawGlycan-SNFG: a robust tool to render glycans and glycopeptides with fragmentation information. *Glycobiology* **2016**, *27*, 200–205.

(S2) Pennington, H. *Escherichia coli* O157. *Lancet* **2010**, *376*, 1428–1435.

(S3) Jorgenson, M. A.; Young, K. D. Interrupting Biosynthesis of O Antigen or the Lipopolysaccharide Core Produces Morphological Defects in *Escherichia coli* by Sequestering Undecaprenyl Phosphate. *J. Bacteriol.* **2016**, *198*, 3070–3079.

(S4) Li, A.; Schertzer, J. W.; Yong, X. Molecular dynamics modeling of *Pseudomonas aeruginosa* outer membranes. *Phys. Chem. Chem. Phys.* **2018**, *20*, 23635–23648.

(S5) Aytenfisu, A. H.; Simon, R.; MacKerell Jr., A. D. Impact of branching on the conformational heterogeneity of the lipopolysaccharide from *Klebsiella pneumoniae*: Implications for vaccine design. *Carbohydr. Res.* **2019**, *475*, 39–47.

(S6) Chen, L.; Mathema, B.; Pitout, J. D.; DeLeo, F. R.; Kreiswirth, B. N. Epidemic *Klebsiella pneumoniae* ST258 is a hybrid strain. *mBio* **2014**, *5*, 10–1128.

(S7) Peterson, A. A.; McGroarty, E. J. High-molecular-weight components in lipopolysaccharides of *Salmonella typhimurium*, *Salmonella minnesota*, and *Escherichia coli*. *J. Bacteriol.* **1985**, *162*, 738–745.

(S8) Pereira, G. P.; Alessandri, R.; Domínguez, M.; Araya-Osorio, R.; Grünewald, L.; Borges-Araújo, L.; Wu, S.; Marrink, S. J.; Souza, P. C.; Mera-Adasme, R. Bartender: Martini 3 Bonded Terms via Quantum Mechanics-Based Molecular Dynamics. *J. Chem. Theory Comput.* **2024**, *20*, 5763–5773.

(S9) Brandner, A. F.; Prakaash, D.; González, A. B.; Waterhouse, F.; Khalid, S. Faster but Not Sweeter: A model of *Escherichia coli* Re-level Lipopolysaccharide for Martini 3 and a Martini 2 Version with Accelerated Kinetics. *J. Chem. Theory Comput.* **2024**, *20*, 6890–6903.

(S10) Vaiwala, R.; Ayappa, K. G. Martini-3 Coarse-Grained Models for the Bacterial Lipopolysaccharide Outer Membrane of *Escherichia coli*. *J. Chem. Theory Comput.* **2023**, *20*, 1704–1716.

(S11) Balusek, C.; Hwang, H.; Lau, C. H.; Lundquist, K.; Hazel, A.; Pavlova, A.; Lynch, D. L.; Reggio, P. H.; Wang, Y.; Gumbart, J. C. Accelerating Membrane Simulations with Hydrogen Mass Repartitioning. *J. Chem. Theory Comput.* **2019**, *15*, 4673–4686.
